# Visceral Adiposity and Inflammation Index as Predictors of Stroke Risk in Middle and Older Age: A Cohort Study Across Blood Pressure Groups

**DOI:** 10.1002/brb3.71147

**Published:** 2025-12-22

**Authors:** Huang Luwen, Yang Linyi, Li Linlin, Chen Ping, Yu Ming

**Affiliations:** ^1^ Department of Neurology Suining Central Hospital Suining Sichuan Province China; ^2^ Department of Pharmacy Suining Central Hospital Suining Sichuan Province China

**Keywords:** CHARLS, hypertension, nonlinear association, stroke, visceral adiposity and inflammation index

## Abstract

**Objective:**

The visceral adiposity and inflammation index (VAII) is a composite index that combines the visceral adiposity index (VAI) and high‐sensitivity C‐reactive protein (hs‐CRP), both of which are associated with stroke risk. This study aimed to investigate the association between VAII and stroke risk, with a particular focus on different blood pressure (BP) statuses, sexes, and age groups.

**Methods:**

Data from 8883 participants in the China Health and Retirement Longitudinal Study were analyzed. VAII levels were categorized into quartiles, and stroke incidence was assessed. Restricted cubic spline analysis was used to assess the association between VAII and stroke risk on the basis of sex, age, and BP status. Sensitivity analyses, including complete‐case analysis, multiple imputation, and calculation of the *E*‐value, were conducted to assess the robustness of the findings.

**Results:**

During a median follow‐up of 9 years, 827 participants (9.31%) developed stroke. In fully adjusted models, a 1‐SD increase in VAII was associated with higher stroke risk (HR = 1.048, 95% CI: 1.009–1.089). Stroke incidence increases across VAII quartiles, with Q4 showing more than twice the risk of Q1 (HR = 2.138, 95% CI: 1.720–2.658). Associations were evident in elevated BP (Q4 vs. Q1: HR = 2.034) and hypertension groups (Q3: HR = 1.906; Q4: HR = 1.713), but not in normal BP. Stronger associations appeared in women and in adults aged 40–60 years. Restricted cubic splines indicated nonlinear relationships. Sensitivity analyses supported the robustness of these findings.

**Conclusion:**

Our study revealed a significant nonlinear relationship between VAII and the risk of stroke. The association between VAII and stroke was nonlinear in individuals with elevated BP and hypertension. Additionally, a nonlinear relationship was observed between VAII and stroke risk in both males and females, as well as in middle‐aged and older adults.

## Graphical abstract

This study identifies a significant nonlinear association between the Visceral Adiposity and Inflammation Index (VAII) and stroke risk in middle‐aged and older adults. The relationship varies by blood pressure status, sex, and age, highlighting VAII as a robust predictor of stroke, particularly in individuals with elevated blood pressure or hypertension.

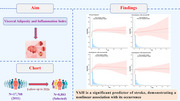

## 1 Introduction

Stroke remains a leading cause of death and long‐term disability worldwide, representing a major public health challenge (GBD 2019 Stroke Collaborators 2021; Ma et al. 2021). In 2019, it was responsible for 6.55 million deaths and 143 million disability‐adjusted life years (GBD 2019 Stroke Collaborators 2021). Despite progress in prevention and treatment, the incidence of stroke continues to rise in low‐ and middle‐income countries (Feigin et al. 2025; Thayabaranathan et al. 2022). Therefore, identifying modifiable risk factors and implementing effective preventive strategies are crucial for primary stroke prevention.

Central adipose tissue consists of subcutaneous adipose tissue and visceral adipose tissue (VAT) compartments (Amato et al. 2013). VAT is particularly harmful because of its infiltration by macrophages and other inflammatory cells, which are associated with increased levels of serum inflammatory markers (Britton et al. 2013; Okamoto et al. 2014). It is also linked to metabolic syndrome and markers of insulin resistance (IR), including elevated fasting insulin levels (Schousboe et al. 2017). VAT can be assessed via abdominal magnetic resonance imaging or computed tomography. However, these methods are expensive and limited, making them unsuitable for large‐scale population screening (Shuster et al. 2012). The visceral adiposity index (VAI) is a widely acknowledged surrogate marker of visceral adipose dysfunction (Amato et al. 2010). Increasing evidence suggests that the VAI is significantly associated with adverse outcomes in atherosclerosis (Yu et al. 2021), stroke (Chen et al. 2022), and cardiovascular disease (Dereziński et al. 2018). High‐sensitivity C‐reactive protein (hs‐CRP) is a powerful indicator of systemic inflammation, is closely linked to stroke risk, and has become an effective biomarker for assessing stroke risk (McCabe et al. 2023). Atherosclerosis is a key risk factor for stroke, with visceral adipose dysfunction and vascular inflammation being central drivers of its pathogenesis (Yang et al. 2023; Xiao et al. 2025). Therefore, developing a comprehensive index reflecting visceral adipose dysfunction and inflammation is essential for stroke prediction. The visceral adiposity and inflammation index (VAII) was first proposed by Zhou et al. (Xiao et al. 2025) and provides a comprehensive reflection of visceral adipose dysfunction and inflammation. Their study demonstrated that the VAII has strong predictive value for stroke incidence. However, despite its potential, VAII has not been widely adopted in clinical research. Furthermore, the nonlinear relationship between VAII and stroke risk has yet to be explored, and its predictive role in individuals with different blood pressure statuses remains inadequately investigated.

To address these gaps, we analyzed data from the China Health and Retirement Longitudinal Study (CHARLS) to examine the relationship between VAII and stroke risk across different blood pressure (BP) statuses. This study aims to facilitate timely interventions for high‐risk individuals, guide targeted prevention strategies, and provide evidence supporting the practical application of VAII in real‐world settings.

## 2 Methods

### 2.1 Study Design

The data for this study were sourced from CHARLS, a national longitudinal survey that tracks individuals aged 45 years and older in China. The first baseline survey (Wave 1) was conducted in 2011 via a multistage stratified sampling method across 28 provinces, and data were collected from 17,708 participants (Zhao et al. 2014). Follow‐up surveys were carried out in 2011, 2013, 2015, 2018, and 2020. All participants provided informed consent, and the study was approved by the Peking University Ethics Committee. The study was conducted in accordance with the principles of the Declaration of Helsinki. The cohort was selected via probability sampling proportional to the population size, achieving an 80.5% response rate, which minimized selection bias and ensured representativeness (Zhao et al. 2014). This sampling strategy ensures that the CHARLS dataset accurately reflects the demographic, socioeconomic, and geographic diversity of China's middle‐aged and older population.

### 2.2 Study Population

In this study, the baseline cohort consisted of participants who completed the 2011 survey and were followed up in the 2020 survey. The exclusion criteria included (1) history of stroke (*n* = 486); (2) missing stroke data (*n* = 182); (3) missing follow‐up data (*n* = 1060); (4) age under 45 years (*n* = 514); (5) missing waist circumference (WC) data (*n* = 3110); (6) missing body mass index (BMI) data (*n* = 129); (7) missing triglyceride (TG) data (n = 3293); (8) missing high‐density lipoprotein cholesterol (HDL‐C) data (n = 3); (9) missing hs‐CRP data (*n* = 1); and (10) missing hypertension data (*n* = 47). The final cohort included 8883 participants (Supplementary Figure S1).

### 2.3 Calculation of VAII

VAII is calculated via the following formula: VAII = VAI × hs‐CRP (mg/L) (Xiao et al. 2025; Chen et al. 2024).

The VAI is calculated via the following formula (Zheng et al. 2022):

$$\begin{array}{ccc} \mathrm{For}\mathrm{males} & : & \left[\mathrm{WC}\left(\mathrm{cm}\right)/\left(39.68+1.88\times \mathrm{BMI}\left(\mathrm{kg}/m^{2}\right)\right)\right]\\ & & \;\times \left(\mathrm{TG}\left(\mathrm{mmol}/L\right)/1.03\right)\times \left(1.31/\mathrm{HDL}\;-C\left(\mathrm{mmol}/L\right)\right) \end{array}$$

$$\begin{array}{ccc} \mathrm{For}\mathrm{females} & : & \left[\mathrm{WC}\left(\mathrm{cm}\right)/\left(36.58\;\times \;1.89\times \mathrm{BMI}\left(\mathrm{kg}/m^{2}\right)\right)\right]\\ & & \times (\mathrm{TG}(\mathrm{mmol}/L)/0.81)\times (1.52/\mathrm{HDL}\;-\;C(\mathrm{mmol}/L)). \end{array}$$

### 2.4 Outcome Definition

The primary outcome of this study was stroke incidence, which was determined by the key question: “Have you ever been diagnosed with a stroke by a physician?” The onset period for stroke was defined as the time between the last interview and the first recorded stroke event (Huo et al. 2025).

### 2.5 Covariates

The covariates in this study included demographic factors (gender, age, residence, education, and marital status), lifestyle factors (smoking, drinking, and sleep duration), physical measurements (height, weight, WC, BMI, and blood pressure), comorbidities [dyslipidemia, hypertension, diabetes mellitus (DM), cancer, pulmonary, heart, liver, kidney, and digestive diseases], and laboratory data [blood urea nitrogen (BUN), serum creatinine (Scr), glucose, total cholesterol (TC), TG, HDL‐C, low‐density lipoprotein cholesterol, and hs‐CRP)].

### 2.6 Definitions

Hypertension was defined by at least one of the following criteria: (1) systolic blood pressure (SBP) ≥ 140 mmHg, (2) diastolic blood pressure (DBP) ≥ 90 mmHg, (3) self‐reported physician diagnosis of hypertension, or (4) use of antihypertensive medications. Elevated BP is defined as an SBP between 120 and 139 mmHg or a DBP between 70 and 89 mmHg (McEvoy et al. 2024). DM is diagnosed on the basis of at least one of the following criteria: (1) fasting blood glucose ≥ 126 mg/dL, (2) hemoglobin A1c ≥ 6.5%, (3) current use of antidiabetic medications, or (4) self‐reported physician diagnosis of DM. Dyslipidemia is identified by one or more of the following: TG ≥ 150 mg/dL, TC ≥ 240 mg/dL, HDL‐C < 40 mg/dL, LDL‐C ≥ 160 mg/dL, current use of lipid‐lowering medications, or self‐reported physician diagnosis of dyslipidemia. Pulmonary diseases include chronic bronchitis, emphysema, and cor pulmonale. Cardiovascular diseases include myocardial infarction, coronary artery disease, angina, congestive heart failure, and other heart conditions (Team CR 2015).

### 2.7 Missing Variables

The distribution of variables with missing data is presented in Supplementary Table S1, where no variable had more than 10% missing values. Multiple imputation with 5 replications was used to retain the maximum sample size, improving the precision and robustness of the analysis.

### 2.8 Statistical Analysis

For quantitative variables following a normal distribution, the results are presented as the means with standard deviations, and between‐group differences were assessed via analysis of variance. For quantitative variables that did not meet the normality assumption, medians and interquartile ranges were calculated, and differences among groups were evaluated via the Kruskal‒Wallis test. Categorical variables are described via counts and percentages, with significant differences evaluated using the chi‐square test.

The participants were stratified into four groups according to the quartiles of VAII. The quartile ranges were as follows: Q1 ≤ 0.76; 0.76 < Q2 ≤ 1.93; 1.93 < Q3 ≤ 5.45; and Q4 > 5.45. The VAII was also analyzed as a continuous variable standardized to one SD to improve robustness. Kaplan‒Meier curves and Gray's test were used to evaluate the incidence of stroke. To investigate potential collinearity between VAII and other covariates, both the generalized variance inflation factor (GVIF) and adjusted GVIF were examined (Supplementary Table S2). The associations between VAII and stroke incidence across different BP statuses, age groups, and sexes were assessed via Cox regression models. Three distinct models were developed for comprehensive analysis: Model 1 was unadjusted; Model 2 included adjustments for sex, age, residence, marital status, education level, smoking status, and drinking status; and Model 3 was further adjusted for sleep duration, cancer, pulmonary disease, heart disease, dyslipidemia, DM, liver disease, and other comorbidities. Additionally, fully adjusted restricted cubic spline (RCS) models with four pre‐specified knots at the fifth, 35th, 65th, and 95th percentiles of VAII were used to assess potential non‐linearity and to explore dose–response relationships between VAII and stroke risk across BP statuses, age groups, and sex categories. To validate the association between VAII and stroke incidence in individuals with different BP statuses, subgroup analyses were conducted. These analyses were performed for individuals with normal BP, elevated BP, and hypertension. Subgroups also included factors such as sex, age, residence, smoking status, drinking status, heart disease, DM, and dyslipidemia.

To increase the reliability of our findings, we performed six robust sensitivity analyses. First, we reanalyzed the dataset after excluding all missing values. Second, we conducted multiple imputations for all missing variables. Second, we conducted an initial MI to address the small proportion of residual missing covariates (<5%) after applying the main exclusion criteria. Third, we performed a full multiple imputation with 20 datasets on the original cohort (*n* = 15,980). The participant inclusion flowchart is shown in Supplementary Figure S2, and the missing‐data summary is provided in Supplementary Table S3. Fourth, to minimize potential reverse causation, we conducted a sensitivity analysis excluding stroke events that occurred within the first two years of follow‐up. Fifth, we calculated the *E*‐value to quantify the minimum strength of association that an unmeasured confounder would need with both VAII and stroke risk to explain the observed associations. The *E*‐value was calculated via the following formula: *E* = *RR* + √(*RR* × (*RR* − 1)) (VanderWeele and Ding 2017). Finally, as an additional assessment of residual confounding, we conducted a negative‐control analysis using “fall injury” as an outcome, which is not biologically linked to VAII. This analysis was performed using the full multiple imputation dataset (20 imputations), applying Cox proportional hazards models with the same covariate adjustments as in the primary analysis.

All analyses were conducted via R statistical software version 4.2.1 (http://www.R‐project.org, The R Foundation) and Free Statistics software version 2.1.1. A *p*‐value of < 0.05 was considered statistically significant.

## 3 Results

### 3.1 Population Characteristics

Baseline data were collected during the first wave of the 2011 survey, involving 8883 participants from the CHARLS dataset, with a mean age of 59 years. A total of 53.74% of the participants were male (Table 1). Among the participants, 2223 (25.03%) had normal BP, 3140 (35.35%) had elevated BP, and 3520 (39.63%) had hypertension. Hypertensive individuals were older (61.65 ± 9.43 years), had a higher BMI (24.19 kg/m^2^), and exhibited higher BP (SBP: 147.22 ± 20.40 mmHg, DBP: 83.26 ± 12.27 mmHg). These patients also had a greater prevalence of comorbidities, including heart disease (16.83%) and dyslipidemia (15.2%) (p < 0.001). Furthermore, individuals with hypertension presented elevated levels of glucose (6.39 ± 2.33 mg/dL), TC (1.34 [0.93, 1.97] mmol/L), and LDL‐C (3.07 ± 0.94 mmol/L). Both the VAI and VAII were significantly greater in this group (*p* < 0.001).

**TABLE 1:** Patient demographics and baseline characteristics.

Characteristics	Total (*n* = 8883)	Normal blood pressure (*n* = 223)	Elevated blood pressure (*n* = 140)	Hypertension (*n* = 3520)	*p‐*value
Age, years	59.32 ± 9.21	57.47 ± 8.69	58.02 ± 8.75	61.65 ± 9.43	< 0.001
Sex (Female), *n* (%)	4774 (53.74)	1237 (55.65)	1595 (50.8)	1942 (55.17)	< 0.001
Residence (Urban), *n* (%)	3080 (34.67)	682 (30.68)	1074 (34.2)	1324 (37.61)	< 0.001
Education level, *n* (%)					< 0.001
No formal education	4274 (48.15)	1101 (49.55)	1393 (44.41)	1780 (50.6)	
Primary school	1950 (21.97)	448 (20.16)	707 (22.54)	795 (22.6)	
Middle school	1772 (19.96)	433 (19.49)	704 (22.44)	635 (18.05)	
High school or above	881 (9.92)	240 (10.8)	333 (10.62)	308 (8.75)	
Current married, *n* (%)	7807 (87.89)	2023 (91)	2824 (89.94)	2960 (84.09)	< 0.001
SBP, mmHg	129.36 ± 21.38	107.49 ± 7.69	124.80 ± 8.47	147.22 ± 20.40	< 0.001
DBP, mmHg	75.31 ± 12.18	62.36 ± 5.61	75.52 ± 6.36	83.26 ± 12.27	< 0.001
Waist circumference, cm	84.30 ± 12.55	80.32 ± 11.50	83.56 ± 12.07	87.48 ± 12.80	< 0.001
BMI, kg/m^2^	23.14 (20.87, 25.78)	21.91 (20.02, 24.04)	22.99 (20.83, 25.45)	24.19 (21.72, 27.03)	< 0.001
Sleep time, hour	6.35 ± 1.88	6.30 ± 1.88	6.44 ± 1.84	6.30 ± 1.92	0.004
Smoking					< 0.001
Never, *n* (%)	5422 (61.04)	1379 (62.03)	1874 (59.68)	2169 (61.64)	
Former, *n* (%)	781 (8.79)	169 (7.6)	255 (8.12)	357 (10.14)	
Current, *n* (%)	2679 (30.16)	675 (30.36)	1011 (32.2)	993 (28.22)	
Drinking					< 0.001
Never, *n* (%)	5237 (58.98)	1326 (59.7)	1824 (58.09)	2087 (59.32)	
Former, *n* (%)	720 (8.11)	174 (7.83)	201 (6.4)	345 (9.81)	
Current, *n* (%)	2922 (32.91)	721 (32.46)	1115 (35.51)	1086 (30.87)	
**Comorbidities**					
DM, *n* (%)	524 (5.95)	76 (3.45)	133 (4.26)	315 (9.03)	< 0.001
Cancer, *n* (%)	75 (0.85)	24 (1.08)	23 (0.73)	28 (0.8)	0.358
Pulmonary disease, *n* (%)	855 (9.65)	205 (9.26)	288 (9.21)	362 (10.3)	0.248
Heart disease, *n* (%)	1029 (11.62)	180 (8.13)	258 (8.24)	591 (16.83)	< 0.001
Dyslipidemia, *n* (%)	831 (9.51)	127 (5.79)	179 (5.79)	525 (15.2)	< 0.001
Liver disease, *n* (%)	292 (3.30)	87 (3.94)	91 (2.91)	114 (3.25)	0.115
Kidney disease, *n* (%)	502 (5.67)	133 (6)	163 (5.22)	206 (5.87)	0.381
Digestive system disease, *n* (%)	2040 (23.00)	629 (28.38)	696 (22.19)	715 (20.33)	< 0.001
**Laboratory parameters**					
BUN, mg/dl	15.75 ± 4.49	15.64 ± 4.37	15.67 ± 4.38	15.88 ± 4.66	0.066
Scr, mg/dl	0.78 ± 0.22	0.75 ± 0.17	0.77 ± 0.18	0.80 ± 0.28	< 0.001
Glucose, mg/dl	6.12 ± 2.02	5.80 ± 1.61	6.05 ± 1.86	6.39 ± 2.33	< 0.001
TC, mmol/L	5.02 ± 1.00	4.85 ± 0.94	5.02 ± 0.99	5.13 ± 1.03	< 0.001
TG, mmol/L	1.20 (0.85, 1.74)	1.05 (0.78, 1.50)	1.15 (0.84, 1.67)	1.34 (0.93, 1.97)	< 0.001
HDL‐C, mmol/L	1.13 ± 0.34	1.18 ± 0.34	1.14 ± 0.34	1.10 ± 0.34	< 0.001
LDL‐C, mmol/L	3.02 ± 0.90	2.89 ± 0.83	3.05 ± 0.90	3.07 ± 0.94	< 0.001
hs‐CRP, mg/L	1.02 (0.55, 2.14)	0.81 (0.47, 1.73)	0.94 (0.53, 1.95)	1.23 (0.65, 2.55)	< 0.001
VAI	1.74 (1.03, 3.06)	1.48 (0.91, 2.43)	1.66 (1.00, 2.84)	2.11 (1.20, 3.70)	< 0.001
VAII	1.93 (0.76, 5.45)	1.26 (0.60, 3.52)	1.66 (0.70, 4.57)	2.82 (1.07, 7.58)	< 0.001

**Abbreviations**: BMI, Body mass index; BUN, blood urea nitrogen; DBP, diastolic blood pressure; DM, diabetes mellitus; HDL‐C, high‐density lipoprotein cholesterol; hs‐CRP, high‐sensitivity C‐reactive protein; LDL‐C, low‐density lipoprotein cholesterol; SBP, systolic blood pressure; Scr, serum creatinine; TC, total cholesterol; TG, triglycerides; VAI, visceral adiposity index; VAII, visceral adiposity and inflammation index.

After a 9‐year follow‐up, a total of 827 (9.31%) participants experienced stroke (Supplementary Table S4). Compared with those without stroke, those with stroke were significantly older (61.35 ± 8.45 years), had higher BP (SBP: 136.90 ± 23.46 mmHg, DBP: 78.63 ± 12.73 mmHg), had a higher BMI (24.06 [21.70, 26.82] kg/m^2^), and had a shorter sleep time (6.17 ± 1.95 h). The stroke group also presented a greater prevalence of DM (9.11%) and heart disease (19.76%) (*p* < 0.001). Laboratory markers, including glucose (6.45 ± 2.41 mg/dL), TC (1.33 [0.96, 1.90] mmol/L), and hs‐CRP (1.33 [0.68, 2.66] mg/L), were significantly elevated in the stroke group (*p* < 0.001). Additionally, both the VAI and VAII were notably greater in stroke patients (*p* < 0.001).

### 3.2 Associations Between VAII Scores and Stroke Risk According to Blood Pressure Status

**FIGURE 1:**
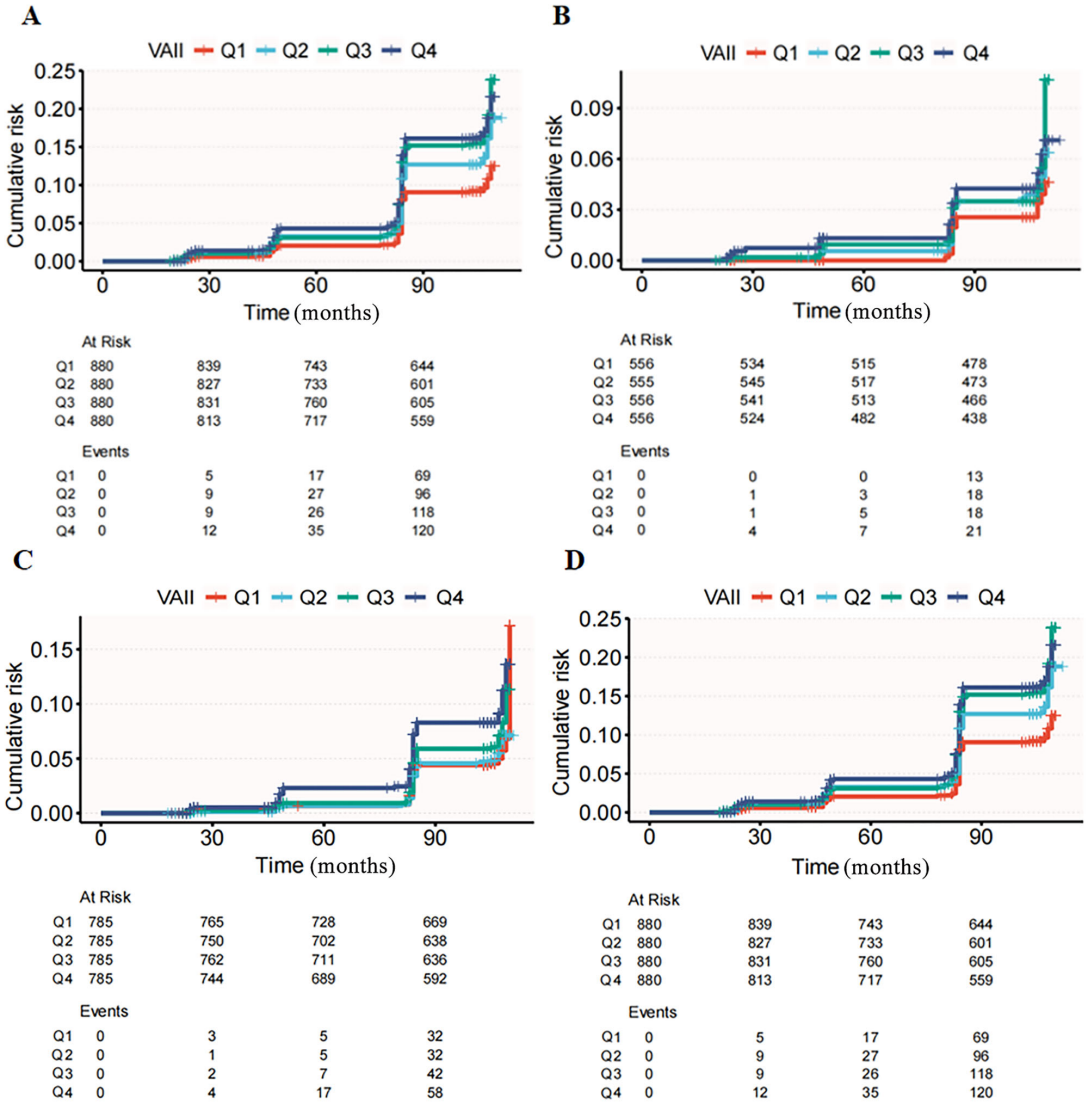
Competing risk model analysis depicting the cumulative incidence of stroke across the VAII quartiles. **(A)** All participants; **(B)** Participants with normal BP; **(C)** Participants with elevated BP; and **(D)** Participants with hypertension.

**FIGURE 2:**
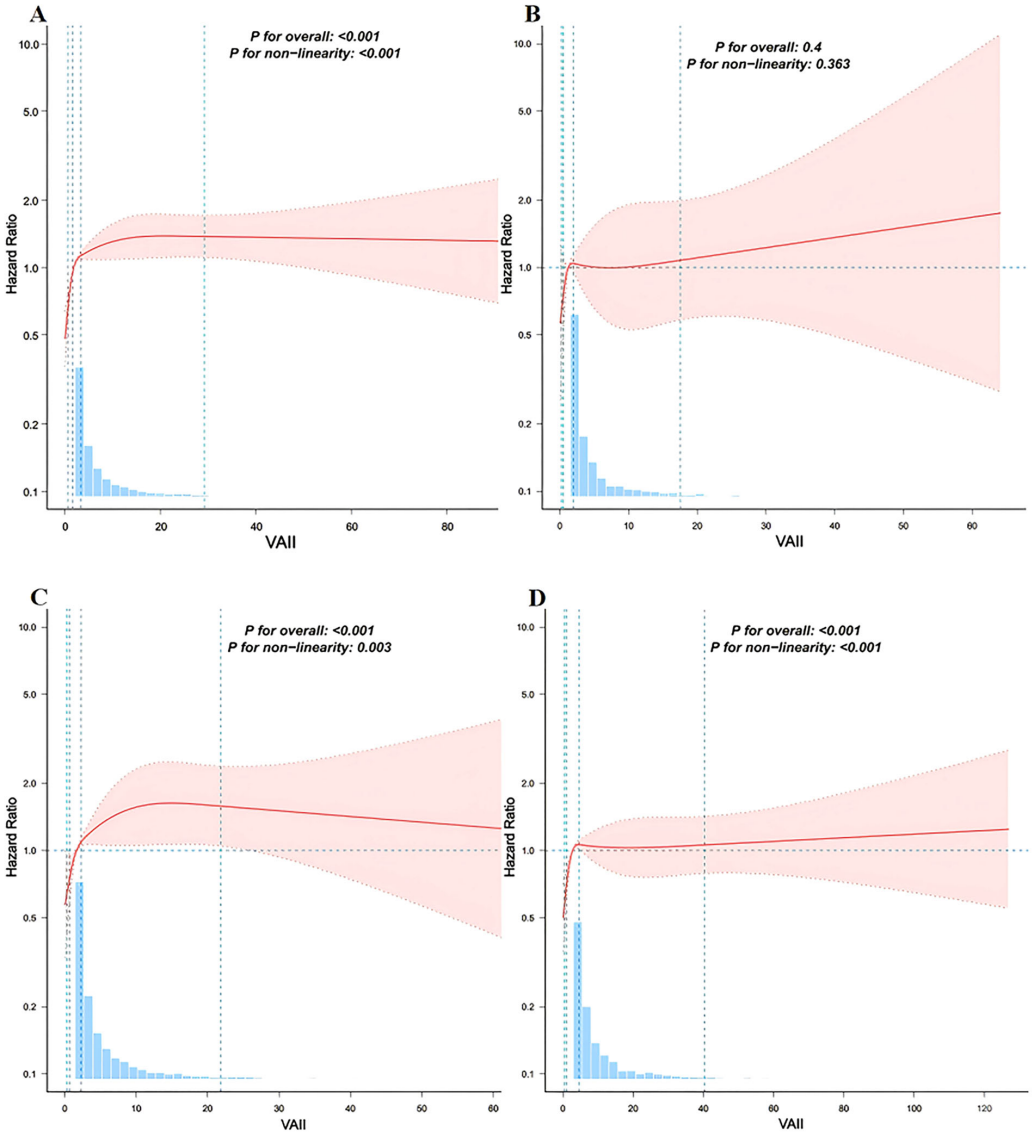
Associations between VAII and the risk of stroke according to blood pressure status. **(A)** All participants; **(B)** Participants with normal BP; **(C)** Participants with elevated BP; and **(D)** Participants with hypertension. Restricted cubic spline models were fitted using four pre‐specified knots at the 5th, 35th, 65th, and 95th percentiles of VAII. The model was adjusted for sex, age, residence, marital status, education level, smoking status, drinking status, sleep duration, DM, cancer status, pulmonary disease, heart disease, dyslipidemia, liver disease, and kidney disease.

In the total population, stroke incidence increased from Q1 to Q4, with 130 cases (5.9%) in Q1, 182 cases (8.2%) in Q2, 236 cases (10.6%) in Q3, and 279 cases (12.6%) in Q4. The cumulative risk plots (Figure 1A) revealed that Q3 had the highest stroke risk, particularly after 90 months. whereas individuals with normal BP showed no such trend (Figure 1B). A similar trend was observed in both the elevated BP group (Figure 1C) and the hypertension group (Figure 1D), with stroke risk in Q3 exceeding that in Q4. In the total population, after adjusting for confounding factors in Model 3, VAII was significantly associated with stroke risk, with each 1‐SD increase in VAII increasing the risk (HR: 1.408, *p* = 0.017) (Table 2). Stroke risk increased progressively across VAII quartiles. In the overall population, individuals in the Q4 had more than twice the risk of stroke compared with those in the Q1 (HR = 2.138, *p* < 0.001). A similar pattern was observed among participants with elevated BP, where Q4 was associated with a higher risk (HR = 2.034, *p* < 0.001). In the hypertension subgroup, both Q3 (HR = 1.906, *p* < 0.001) and Q4 (HR = 1.713, *p* < 0.001) were associated with significantly increased stroke risk relative to Q1. However, no significant associations were found in the normal BP group. RCS analysis revealed a nonlinear relationship between VAII and stroke events in the total population, elevated BP, and hypertension groups (Figures 2A,  2C, and  2D) (*P* for nonlinearity < 0.001). No significant association was found in the normal BP group (Figure 2B).

**TABLE 2:** Association between VAII and the risk of stroke according to blood pressure status.

Categories	Characteristic	Event, (*n*%)	Model 1		Model 2		Model 3	
			HR (95% CI)	*p*‐value	HR (95% CI)	*p*‐value	HR (95% CI)	*p*‐value
Total	VAII (per 1 SD)	827 (9.3)	1.053 (1.019, 1.087)	0.002	1.052 (1.018, 1.087)	0.002	1.048 (1.009, 1.089)	0.017
	VAII							
	Q1	130 (5.9)	1(Ref)		1(Ref)		1(Ref)	
	Q2	182 (8.2)	1.458 (1.164, 1.826)	0.001	1.427 (1.138, 1.789)	0.002	1.391 (1.105, 1.75)	0.005
	Q3	236 (10.6)	1.926 (1.555, 2.386)	< 0.001	1.883 (1.516, 2.338)	< 0.001	1.805 (1.448, 2.25)	< 0.001
	Q4	279 (12.6)	2.382 (1.934, 2.933)	< 0.001	2.326 (1.882, 2.875)	< 0.001	2.138 (1.72, 2.658)	< 0.001
	P for trend		1.323 (1.243, 1.409)	< 0.001	1.314 (1.233, 1.401)	< 0.001	1.279 (1.197, 1.366)	< 0.001
Normal BP	VAII (per 1 SD)	109 (4.9)	1.071 (0.936, 1.225)	0.318	1.084 (0.943, 1.246)	0.257	1.189 (0.949, 1.49)	0.132
	VAII							
	Q1	20 (3.6)	1 (Ref)		1 (Ref)		1 (Ref)	
	Q2	26 (4.7)	1.316 (0.735, 2.358)	0.356	1.275 (0.71, 2.29)	0.416	1.338 (0.742, 2.412)	0.333
	Q3	33 (5.9)	1.69 (0.97, 2.944)	0.064	1.696 (0.969, 2.968)	0.065	1.668 (0.95, 2.928)	0.075
	Q4	30 (5.4)	1.637 (0.93, 2.883)	0.088	1.579 (0.89, 2.802)	0.119	1.494 (0.827, 2.7)	0.184
	P for trend		1.18 (0.995, 1.399)	0.057	1.171 (0.986, 1.391)	0.072	1.146 (0.96, 1.367)	0.131
Elevated BP	VAII (per 1 SD)	227 (7.2)	1.025 (0.951, 1.103)	0.519	1.019 (0.946, 1.098)	0.626	1.027 (0.956, 1.104)	0.462
	VAII							
	Q1	43 (5.5)	1 (Ref)		1 (Ref)		1 (Ref)	
	Q2	46 (5.9)	1.116 (0.736, 1.691)	0.606	1.105 (0.728, 1.678)	0.640	1.109 (0.722, 1.703)	0.637
	Q3	61 (7.8)	1.472 (0.997, 2.175)	0.052	1.5 (1.01, 2.229)	0.045	1.518 (1.012, 2.277)	0.044
	Q4	77 (9.8)	1.973 (1.359, 2.866)	< 0.001	2.045 (1.395, 2.998)	< 0.001	2.034 (1.376, 3.007)	< 0.001
	P for trend		1.269 (1.127, 1.429)	< 0.001	1.288 (1.14, 1.455)	< 0.001	1.286 (1.136, 1.457)	< 0.001
Hypertension	VAII (per 1 SD)	491 (13.9)	1.063 (1.001, 1.128)	0.048	1.061 (1.001, 1.125)	0.048	1.041 (0.975, 1.111)	0.227
	VAII							
	Q1	82 (9.3)	1 (Ref)		1 (Ref)		1 (Ref)	
	Q2	120 (13.6)	1.507 (1.138, 1.996)	0.004	1.483 (1.118, 1.968)	0.006	1.388 (1.04, 1.854)	0.026
	Q3	150 (17)	1.866 (1.425, 2.442)	< 0.001	1.906 (1.451, 2.503)	< 0.001	1.79 (1.355, 2.364)	< 0.001
	Q4	139 (15.8)	1.833 (1.395, 2.408)	< 0.001	1.885 (1.428, 2.489)	< 0.001	1.713 (1.286, 2.281)	< 0.001
	P for trend		1.207 (1.114, 1.308)	< 0.001	1.223 (1.126, 1.328)	< 0.001	1.191 (1.093, 1.297)	< 0.001

Model 1: unadjusted for any covariates Model 2: adjusted for gender, age, residence, marital status, education level, smoking status, and drinking status Model 3: adjusted for gender, age, residence, marital status, education level, smoking, drinking, sleep time, DM, cancer, pulmonary disease, heart disease, dyslipidemia, liver disease, and kidney disease. **Abbreviations**: BP, blood pressure; SD, standard deviation; VAII, Visceral adiposity and inflammation index.

### 3.3 Associations Between VAII Scores and Stroke Risk According to Age and Sex

**FIGURE 3:**
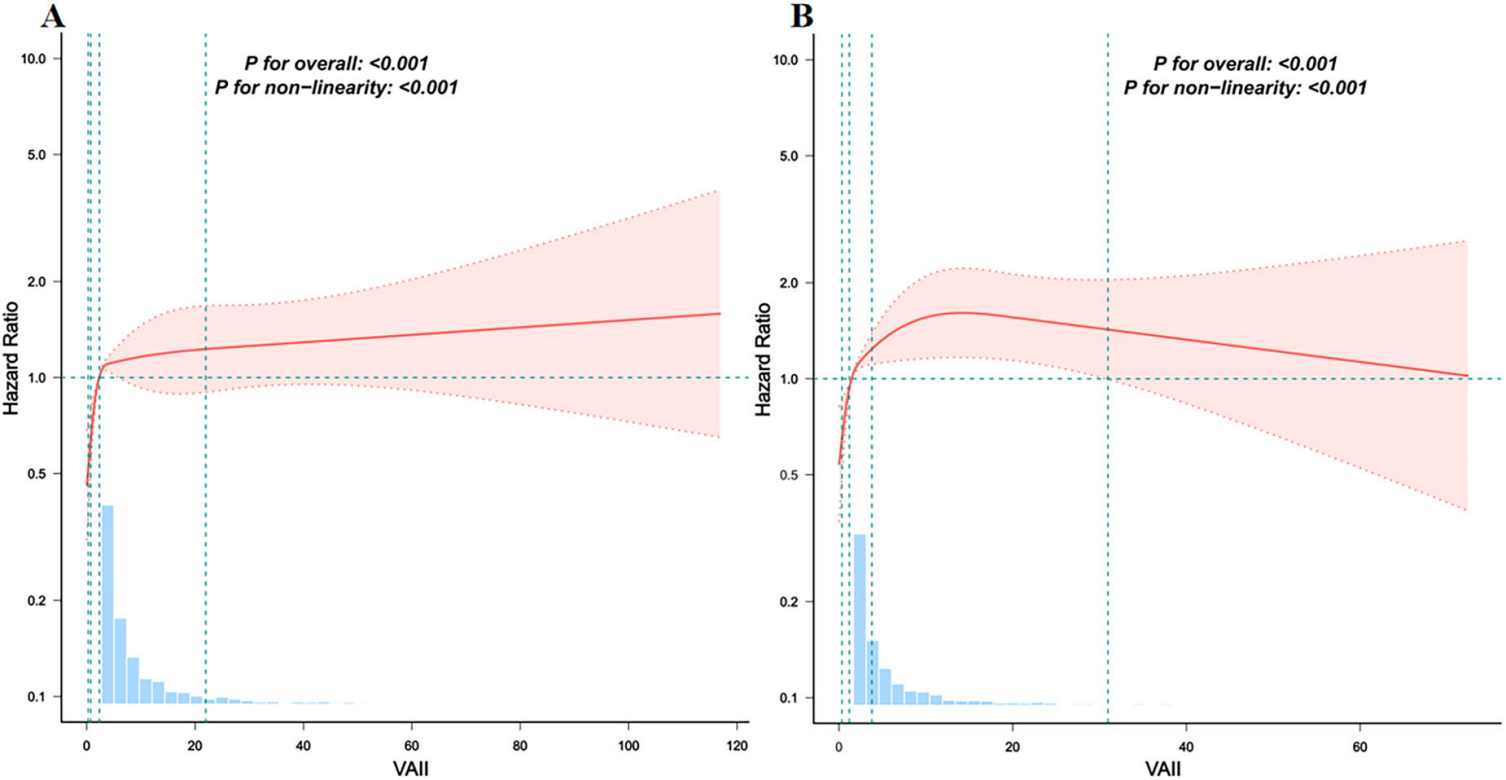
Associations between VAII scores and the risk of stroke according to sex. **(A)** Male; and **(B)** Female. Restricted cubic spline models were fitted using four pre‐specified knots at the 5th, 35th, 65th, and 95th percentiles of VAII. The model was adjusted for sex, age, residence, marital status, education level, smoking status, drinking status, sleep duration, DM, cancer status, pulmonary disease, heart disease, dyslipidemia, liver disease, and kidney disease.

In sex‐stratified analyses, VAII showed a modest association with stroke risk among women (Table 3). In the fully adjusted model, the per‐SD increase in VAII demonstrated a borderline, non‐significant trend toward higher stroke risk (HR = 1.068, *p* = 0.054). When VAII was analyzed categorically, stroke risk increased across quartiles, with women in Q4 having higher risk compared with Q1 (HR = 2.169, 95% CI: 1.595–2.949). Among men, VAII was not significantly associated with stroke risk on a per‐SD basis. However, in the quartile analysis, higher quartiles were associated with increased risk, with Q3 (HR = 1.708, *p* < 0.001) and Q4 (HR = 2.105, *p* < 0.001) showing higher risk relative to Q1. Additionally, RCS analysis revealed a significant nonlinear relationship between VAII and stroke incidence in both males and females (Figures 3A and 3B).

**TABLE 3:** Association between VAII and the risk of stroke according to gender.

Sex	Characteristic	Event, (*n*%)	Model 1		Model 2		Model 3	
			HR (95% CI)	*p‐*value	HR (95% CI)	*p‐*value	HR (95% CI)	*p‐*value
Male	VAII (per 1 SD)	393 (9.6)	1.038 (0.991, 1.089)	0.118	1.04 (0.991, 1.091)	0.114	1.037 (0.984, 1.093)	0.17
	VAII							
	Q1	64 (6.2)	1 (Ref)		1 (Ref)		1 (Ref)	
	Q2	79 (7.7)	1.281 (0.921, 1.782)	0.141	1.248 (0.897, 1.736)	0.188	1.199 (0.857, 1.678)	0.29
	Q3	115 (11.2)	1.898 (1.398, 2.576)	< 0.001	1.806 (1.326, 2.458)	< 0.001	1.708 (1.247, 2.34)	< 0.001
	Q4	135 (13.1)	2.361 (1.754, 3.179)	< 0.001	2.313 (1.714, 3.121)	< 0.001	2.105 (1.548, 2.863)	< 0.001
	P for trend		1.343 (1.226, 1.471)	< 0.001	1.335 (1.217, 1.465)	< 0.001	1.296 (1.178, 1.425)	< 0.001
Female	VAII (per 1 SD)	434 (9.1)	1.084 (1.028, 1.143)	0.003	1.077 (1.019, 1.137)	0.008	1.068 (0.999, 1.143)	0.054
	VAII							
	Q1	64 (5.4)	1(Ref)		1(Ref)		1(Ref)	
	Q2	101 (8.5)	1.644 (1.202, 2.248)	0.002	1.537 (1.123, 2.104)	0.007	1.532 (1.111, 2.114)	0.009
	Q3	120 (10.1)	2.004 (1.48, 2.714)	< 0.001	1.803 (1.329, 2.447)	< 0.001	1.825 (1.336, 2.492)	< 0.001
	Q4	149 (12.5)	2.619 (1.954, 3.511)	< 0.001	2.323 (1.727, 3.124)	< 0.001	2.169 (1.595, 2.949)	< 0.001
	P for trend		1.344 (1.233, 1.466)	< 0.001	1.295 (1.186, 1.414)	< 0.001	1.266 (1.155, 1.386)	< 0.001

Model 1: unadjusted for any covariates Model 2: adjusted for sex, age, residence, marital status, education level, smoking status, and drinking status Model 3: adjusted for sex, age, residence, marital status, education level, smoking, drinking, sleep time, DM, cancer, pulmonary disease, heart disease, dyslipidemia, liver disease, and kidney disease. **Abbreviations**: SD, standard deviation; VAII, visceral adiposity index and inflammation index.

**FIGURE 4:**
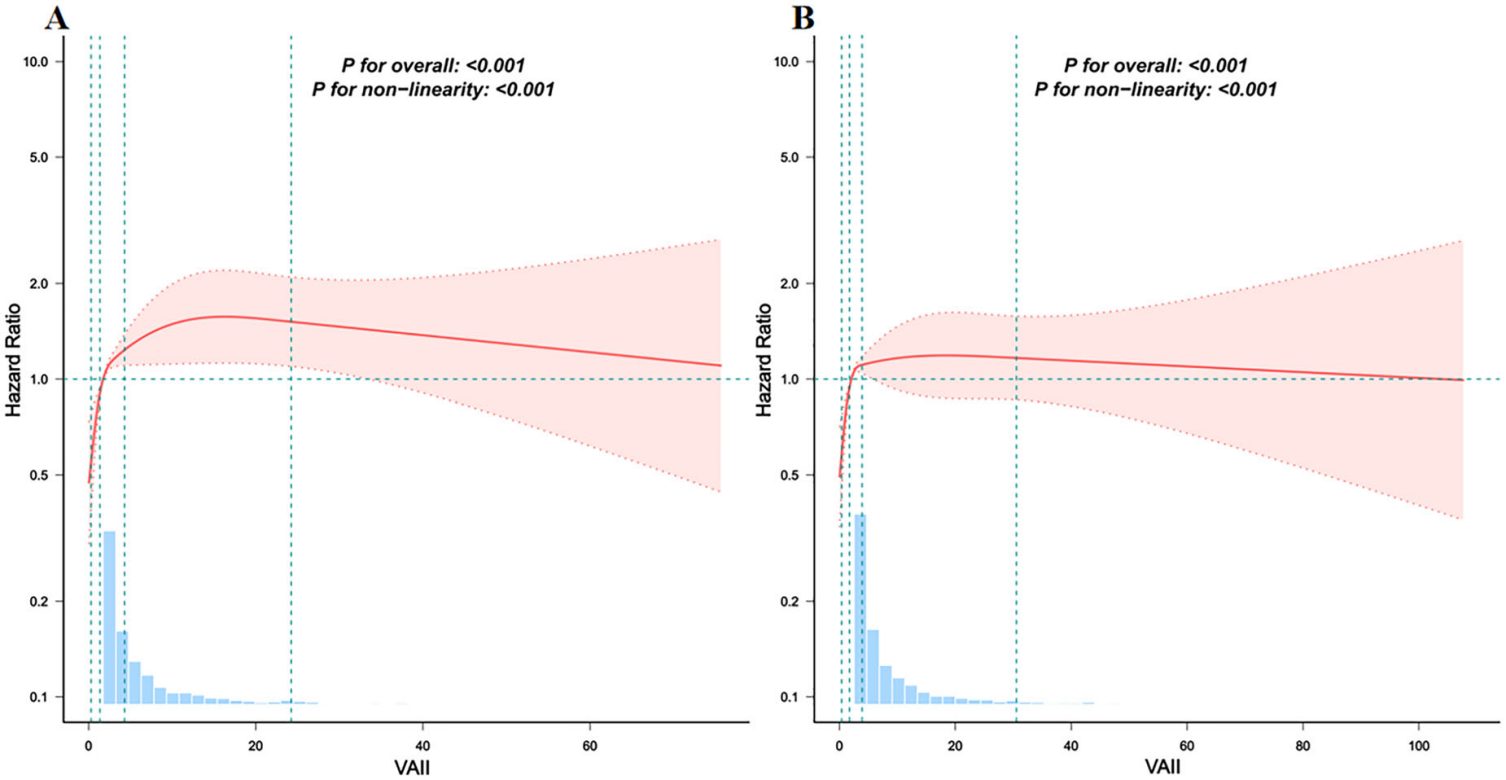
Associations between VAII scores and the risk of stroke according to age. **(A)** Age 45–60; and **(B)** Age ≥ 60. Restricted cubic spline models were fitted using four pre‐specified knots at the 5th, 35th, 65th, and 95th percentiles of VAII. The model was adjusted for sex, age, residence, marital status, education level, smoking status, drinking status, sleep duration, DM, cancer status, pulmonary disease, heart disease, dyslipidemia, liver disease, and kidney disease.

As shown in Table 4, VAII showed a clearer association with stroke risk among adults aged 45–60 years. In the fully adjusted model, the per‐SD increase in VAII demonstrated a non‐significant trend toward higher stroke risk (HR = 1.042, *p* = 0.102), whereas the quartile analysis showed a graded increase in risk. Compared with Q1, stroke risk was higher in Q2 (HR = 1.586, *p* = 0.011), Q3 (HR = 1.869, *p* < 0.001) and Q4 (HR = 2.430, *p* < 0.001). Among individuals aged ≥ 60 years, the association between VAII and stroke risk was more modest. The per‐SD association was not statistically significant (HR = 1.057, *p* = 0.148). However, in the quartile analysis, higher VAII remained associated with increased stroke risk, with Q3 (HR = 1.650, *p* < 0.001), and Q4 (HR = 1.827, *p* < 0.001) demonstrating elevated risks relative to Q1. Additionally, RCS analysis revealed a significant nonlinear relationship between VAII and stroke events in both middle‐aged and older participants (Figures 4A and 4B).

**TABLE 4:** Association between VAII and the risk of stroke according to age.

Age	Characteristic	Event, (*n*%)	Model 1		Model 12		Model 3	
			HR (95% CI)	*p‐*value	HR (95%CI)	*p‐*value	HR (95% CI)	*p‐*value
40–60	VAII (per 1 SD)	358 (7.4)	1.05 (1.006, 1.095)	0.026	1.049 (1.006, 1.095)	0.026	1.042 (0.992, 1.094)	0.102
	VAII							
	Q1	51 (4.2)	1 (Ref)		1(Ref)		1(Ref)	
	Q2	79 (6.5)	1.571 (1.105, 2.235)	0.012	1.596 (1.122, 2.272)	0.009	1.586 (1.111, 2.265)	0.011
	Q3	97 (8)	1.977 (1.409, 2.775)	< 0.001	2.03 (1.443, 2.857)	< 0.001	1.869 (1.321, 2.646)	< 0.001
	Q4	131 (10.8)	2.734 (1.978, 3.779)	< 0.001	2.814 (2.03, 3.903)	< 0.001	2.43 (1.735, 3.403)	< 0.001
	P for trend		1.373 (1.247, 1.512)	< 0.001	1.385 (1.257, 1.527)	< 0.001	1.312 (1.187, 1.45)	< 0.001
≥ 60	VAII (per 1 SD)	469 (11.7)	1.074 (1.007, 1.145)	0.03	1.07 (1.001, 1.144)	0.047	1.057 (0.98, 1.14)	0.149
	VAII							
	Q1	86 (8.6)	1(Ref)		1(Ref)		1(Ref)	
	Q2	102 (10.2)	1.245 (0.934, 1.659)	0.134	1.253 (0.939, 1.672)	0.125	1.223 (0.909, 1.645)	0.183
	Q3	135 (13.4)	1.673 (1.276, 2.192)	< 0.001	1.652 (1.255, 2.173)	< 0.001	1.65 (1.247, 2.184)	< 0.001
	Q4	146 (14.5)	1.928 (1.477, 2.516)	< 0.001	1.924 (1.463, 2.531)	<0.001	1.827 (1.377, 2.425)	< 0.001
	P for trend		1.251 (1.152, 1.358)	< 0.001	1.248 (1.146, 1.358)	< 0.001	1.23 (1.127, 1.343)	< 0.001

Model 1: unadjusted for any covariates Model 2: adjusted for sex, age, residence, marital status, education level, smoking status, and drinking status Model 3: adjusted for sex, age, residence, marital status, education level, smoking, drinking, sleep time, DM, cancer, pulmonary disease, heart disease, dyslipidemia, liver disease, and kidney disease. **Abbreviations**: VAII, visceral adiposity index and inflammation index; SD, standard deviation.

### 3.4 Subgroup Analysis by Blood Pressure Status

**FIGURE 5:**
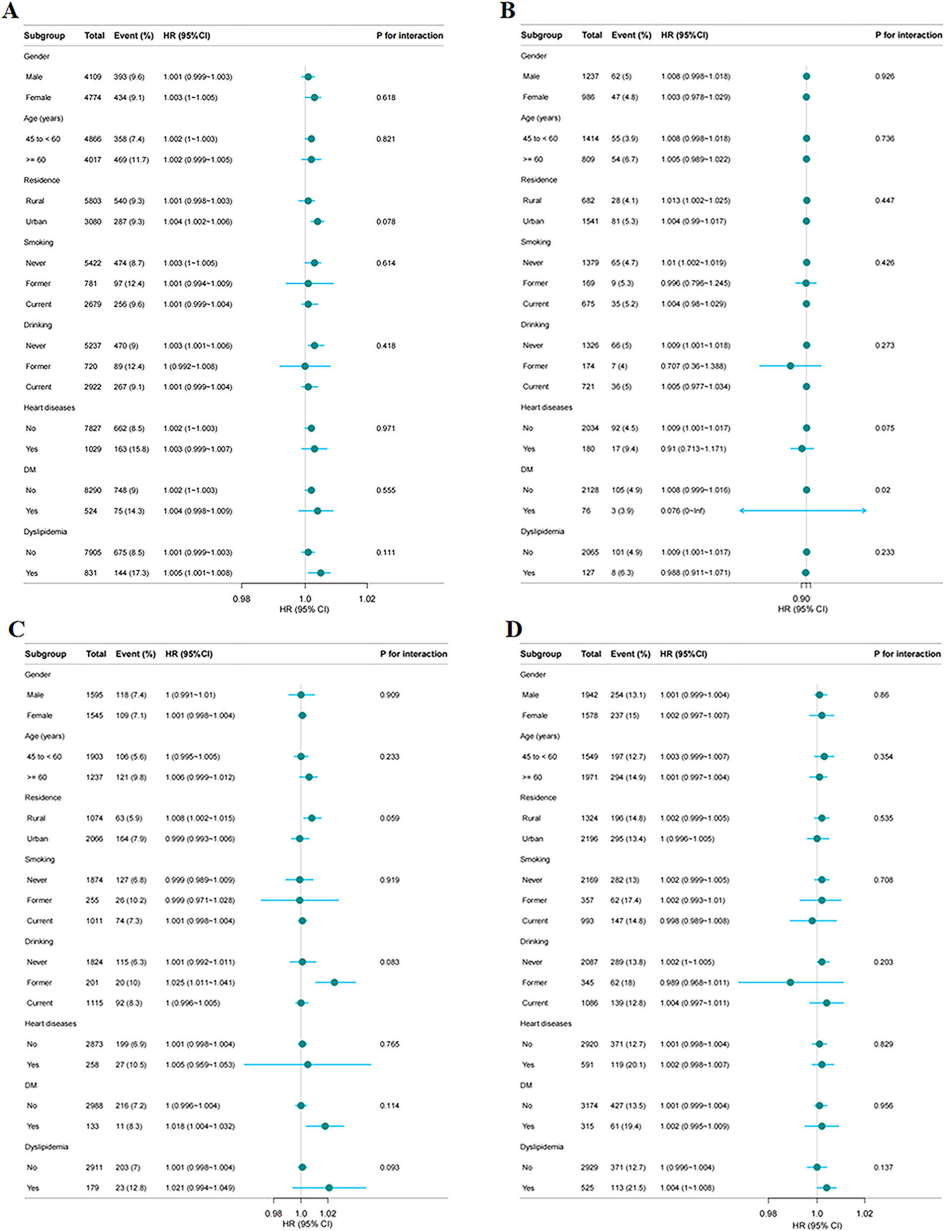
Subgroup analyses of individuals with different blood pressure statuses were performed to investigate the relationship between VAII and hypertension across various subgroups. **(A)** All participants; **(B)** Participants with normal BP; **(C)** Participants with elevated BP; and **(D)** Participants with hypertension. The model was adjusted for sex, age, residence, marital status, education level, smoking status, drinking status, sleep duration, DM status, cancer status, pulmonary disease status, heart disease status, dyslipidemia status, liver disease status, and kidney disease status.

Subgroup analyses were conducted to assess the applicability of the VAII across different BP statuses, considering variables such as sex, age, residence, smoking, drinking, heart disease, DM, and dyslipidemia. In the normal BP group, a significant interaction between DM and VAII was observed (interaction *p* = 0.02) (Figure 5B). In the overall population (Figure 5A), the elevated BP group (Figure 5C), and the hypertension group (Figure 5D), no significant interactions were found (*p* > 0.05).

### 3.5 Sensitivity Analysis

To evaluate the robustness of the study findings, several sensitivity analyses were conducted. First, after all missing data were excluded, the results did not change substantially (Supplementary Tables S5, S6, and S7). Second, both the initial multiple imputation for residual missing values and the full multiple imputation with 20 imputations yielded effect estimates that were consistent with the primary analysis (Supplementary Tables S8–S11). Third, excluding stroke events in the first two years of follow‐up did not materially alter the association (Supplementary Table S12), suggesting that early subclinical cerebrovascular disease is unlikely to explain the findings. Fourth, the E value for VAII, calculated from Model 3, was 3.7. This finding indicates that a relatively large unmeasured confounding effect could explain the observed risk ratio (Supplementary Figure S3). Finally, in the negative‐control analysis using fall injury as the outcome, VAII showed no overall association with fall risk in the total population or any BP subgroup (Supplementary Table S13). Although two isolated quartile comparisons reached nominal significance, these estimates were small, inconsistent in direction, and not reproduced in adjacent categories or models, indicating that they are likely due to chance.

## 4 Discussion

This large‐scale study revealed a significant association between VAII and stroke risk, with the relationship evaluated across different blood pressure statuses, sexes, and age groups. Our results demonstrate a notable nonlinear association between VAII and stroke incidence. Elevated VAII levels were linked to a nonlinear relationship with stroke risk in individuals with elevated BP and hypertension. However, no such association was observed in individuals with normal BP. The nonlinear relationship was similar across both genders and consistent in middle‐aged and older participants. These findings highlight the complexity of the relationship between VAII and stroke risk and underscore the importance of examining this association within specific BP status. Consequently, implementing tailored assessment strategies for different populations will be more effective in identifying high‐risk individuals.

The VAII is a composite index that integrates the VAI, a marker of visceral adipose dysfunction, and hs‐CRP, a biomarker of systemic inflammation. Substantial evidence supports strong associations between each component of the VAII and cerebrovascular risk. Elevated VAI levels have been consistently linked to the incidence of stroke, earlier stroke onset, silent brain infarction, and stroke mortality across multiple cohorts and study designs (Chen et al. 2022; Dereziński et al. 2018; Nam et al. 2020; Chang et al. 2023; Herlina et al. 2024; Amato et al. 2011; Han et al. 2014; Park et al. 2016; Cui et al. 2022). Likewise, inflammation plays a central role in stroke pathophysiology, and higher hs‐CRP concentrations have been associated with increased stroke mortality, recurrence, and poor prognosis in large meta‐analyses, with Mendelian randomization studies further supporting a causal relationship between CRP and stroke risk (Zhou et al. 2016; Chen et al. 2023; Yang et al. 2024). In addition, recent work has shown that co‐exposure to metabolic dysfunction (reflected by VAI) and inflammation (reflected by CRP) further amplifies stroke risk (Xiao et al. 2025). Because VAI reflects visceral fat–related metabolic impairment while hs‐CRP captures systemic inflammatory burden, combining these two pathways provides a more comprehensive representation of an individual's metabolic–inflammatory state (Amato and Giordano 2014). Accordingly, VAII may serve as an integrated risk marker that captures complementary biological processes relevant to stroke susceptibility.

In our study, we analyzed data from 8,883 participants in the CHARLS database and reported a nonlinear association between elevated VAII levels and stroke incidence. In individuals with elevated BP and hypertension, a nonlinear relationship between VAII levels and stroke risk persisted, but no such association was observed in those with normal BP. This may be explained by the fact that individuals with hypertension already have significant vascular sclerosis and endothelial dysfunction. These conditions make the vascular system more responsive to changes in VAII, which can trigger more severe vascular damage and inflammation, thereby increasing stroke risk. Once VAII reaches a certain threshold, the vascular system may have already sustained considerable damage, reducing the impact of further increases in VAII. In contrast, individuals with elevated BP but not yet diagnosed with hypertension may be in a transitional state. This makes their vascular system more susceptible to changes in VAII, resulting in a greater inflection point for this group. Those with normal BP generally have healthier and more stable vascular systems, making them less sensitive to changes in VAII; thus, no significant association was detected. Therefore, this study emphasizes the need to monitor VAII levels in patients while considering their BP status. Additionally, we observed sex differences. In Model 1 and Model 2, stroke risk in women was significantly greater than that in men at lower to moderate VAII levels. However, in the fully adjusted Model 3, the risk response was similar between men and women. A nonlinear relationship between VAII and stroke risk exists in both men and women. Previous studies have shown that men generally have higher stroke incidence and mortality rates between the ages of 45 and 74, but this trend is reversed in individuals aged 74 and older (Ospel et al. 2023; Reeves et al. 2008). This shift may be because women, with their longer life expectancy, tend to live longer with risk factors that increase stroke susceptibility at an older age, leading to a convergence in stroke risk responses between genders in older populations (Rexrode et al. 2022). This may explain why sex differences in stroke risk associated with VAII become less significant after adjusting for other factors in middle‐aged and elderly populations. Therefore, to effectively reduce stroke risk, clinicians should monitor VAII levels in both men and women. The association between VAII and stroke risk was consistent across age groups, but the HR was greater for individuals aged 45–60 years. This phenomenon may be closely related to the unique metabolic and physiological changes in middle‐aged individuals. For example, middle‐aged individuals may present with metabolic syndrome (Kim et al. 2022), IR (Castro et al. 2023), and increased chronic inflammation (Nersesian et al. 2018). Therefore, VAII levels in individuals aged 45–60 years should be prioritized to reduce stroke risk.

We conducted a comprehensive analysis of the relationships among BP status, sex, age, and stroke risk. The results revealed a similar association between VAII and stroke risk in both men and women with hypertension, emphasizing the importance of maintaining lower VAII levels in this group. Stroke risk was significantly greater in both men and women with elevated BP in Q4 than in those with elevated BP in Q1, highlighting the need to monitor VAII levels in this group. Further analysis revealed that elevated VAII levels were significantly associated with increased stroke risk in both middle‐aged patients with hypertension and elderly individuals with elevated BP or hypertension. These findings underscore the importance of reducing VAII levels to lower stroke incidence. Additionally, in individuals with normal BP, the subgroup analysis revealed a significant interaction effect between VAII levels and DM, which may be due to the low proportion of diabetic patients, resulting in an underestimation of their stroke risk and a lower HR.

Although the exact mechanisms linking VAII to stroke risk remain unclear, several potential pathways can be proposed. First, visceral fat, a highly active metabolic tissue, not only serves as an energy reserve but also secretes various cytokines and hormones, including tumor necrosis factor and interleukin‐6. These molecules can promote systemic low‐grade chronic inflammation (Kolb 2022; Kawai et al. 2021), which is considered a significant risk factor for stroke, particularly ischemic stroke (Du et al. 2011). Second, excessive visceral fat and the accompanying inflammatory response contribute to the development of IR, exacerbating metabolic disturbances and increasing stroke risk (Bensussen et al. 2023). Third, the accumulation of visceral fat promotes lipid infiltration into vascular walls, accelerating the progression of atherosclerosis (Wu et al. 2025). Inflammation may also destabilize atherosclerotic plaques, increasing the risk of plaque rupture and, consequently, stroke (Li et al. 2025). Additionally, visceral fat may alter plasma viscosity and blood rheology, increasing blood viscosity, impeding circulation, and creating conditions conducive to thrombosis, further increasing stroke risk (Brun et al. 2016). Therefore, it can be reasonably hypothesized that individuals with higher VAII levels may experience more severe vascular damage, resulting in increased stroke risk. However, the underlying mechanisms require further investigation.

This study presents several innovations. First, using data from the CHARLS database, we are the first to examine the association between VAII and stroke incidence across BP status, sex, and age. Second, we identified a dose‒response relationship between VAII and stroke risk in these different groups. This finding has significant clinical implications for the early identification and prevention of high‐risk individuals. Finally, our subgroup analyses revealed consistent results across various population characteristics, providing valuable insights for clinical practice. Additionally, VAII is easily measurable, enhancing its practical application in clinical settings.

However, this study has several limitations. First, stroke diagnoses were self‐reported based on healthcare provider assessments and may therefore be subject to misclassification. Prior studies suggest that the sensitivity and specificity of self‐reported stroke are only moderate, indicating that some degree of non‐differential error is likely. Future studies incorporating full medical‐record linkage will be important for improving diagnostic accuracy (Tack et al. 2025; Choe et al. 2019). Second, the CHARLS questionnaire lacks stroke subtype classification, limiting the ability to assess risks for different stroke types. Future studies should collect more detailed data to address this gap. Third, although we adjusted for a wide range of potential confounders, the possibility of residual confounding cannot be entirely excluded. Several lifestyle‐related variables—such as diet quality, sodium intake, and physical activity—were not available in CHARLS, and these factors typically cluster with adverse metabolic profiles. Their absence could therefore bias the VAII–stroke association toward a stronger positive direction, although the magnitude of such bias is difficult to estimate. The *E*‐value calculated in our study suggests that an unmeasured confounder would need to have a relatively strong association with both VAII and stroke to fully account for the observed results. However, the *E*‐value addresses only confounders independent of measured covariates and does not address systematic measurement error. To further evaluate residual bias, we incorporated a negative‐control outcome analysis using fall injuries, which are not biologically related to VAII. The lack of association between VAII and fall risk in both the overall population and blood pressure subgroups supports the interpretation that substantial residual confounding or model‐driven bias is unlikely to fully explain the observed associations. Fourth, the study was conducted among middle‐aged and older Chinese adults, which may limit the broader generalizability of the findings. Further research is needed to determine whether the associations we observed extend to younger groups or to individuals from other ethnic backgrounds. In addition, multicenter prospective studies could help further confirm the applicability of VAII, and intervention studies could explore whether reducing VAII levels in high‐risk groups may lower subsequent stroke risk.

## 5 Conclusion

VAII is a significant predictor of stroke, showing a nonlinear association with stroke incidence. This nonlinear relationship was observed in individuals with elevated blood pressure and hypertension but not in those with normal BP. A similar nonlinear association between VAII and stroke risk was found across different sexes and age groups. These findings emphasize the need for tailored risk management strategies based on sex, age, and BP status to prevent stroke in at‐risk individuals.

## Author Contributions

**Huang Luwen**: conceptualization, methodology, data curation, formal analysis, and writing – original draft. **Yang Linyi**: conceptualization, methodology, formal analysis, and writing – original draft. **Li Linlin**: conceptualization, methodology, and visualization. **Chen Ping**: methodology, formal analysis, visualization, writing – review and editing. **Yu Ming**: conceptualization, methodology, supervision, writing – review and editing.

## Funding

The authors have nothing to report.

## Ethics Statement

The CHARLS study was approved by the Biomedical Ethics Review Committee of Peking University (IRB00001052–11015).

## Consent

A written informed consent was obtained from all participants.

## Conflicts of Interest

The authors declare that they have competing interests.

## Supporting information



Table S1 Distribution of variables with missing data
Table S2 Assessment of collinearity among independent variables in the final regression model
Table S3 Distribution of variables with missing data in all populations
Table S4 Characteristics of the population that developed stroke and those without, based on data from 2011
Table S5 Characteristics of the population that developed stroke and those without, based on data from 2011
Table S6 Association between VAII and the risk of stroke according to gender after excluding missing data
Table S7 Association between VAII and the risk of stroke according to age after excluding missing data
Table S8 Association between VAII and stroke risk according to blood pressure regulation status after multiple imputation
Table S9 Association between VAII and the risk of stroke according to gender after multiple imputation
Table S10 Association between VAII and the risk of stroke according to age after multiple imputation
Table S11 Association between VAII and stroke risk by blood pressure status full‐cohort multiple imputation
Table S12 Association between VAII and stroke risk by blood pressure status after excluding stroke events within the first two years of follow‐up
Table S13 Association of VAII with fall injury across blood pressure status after full‐cohort multiple imputation
Figure S1 Flowchart of the study population.
Figure S2 Flowchart of the study population following full multiple imputation with 20 datasets in the original cohort.
Figure S3 *E*‐value plot evaluating the risk ratios of VAII in relation to stroke risk. Adjusted for sex, age, residence, marital status, education level, smoking, drinking, sleep time, DM, cancer, pulmonary disease, heart disease, dyslipidemia, liver disease, and kidney disease.

## Data Availability

The CHARLS datasets are available for download at the CHARLS home website (http://charls.pku.edu.cn/en).
