# Supplementary material for: Visceral Adiposity and Inflammation Index as Predictors of Stroke Risk in Middle and Older Age: A Cohort Study Across Blood Pressure Groups
Source: Brain Behav. 2025 Dec 22;15(12):e71147. doi: 10.1002/brb3.71147 (PMC12723208; doi:10.1002/brb3.71147)
Supplement: Supplementary file 1 — Table S1 Distribution of variables with missing data Table S2 Assessment of collinearity among independent variables in the final regression model Table S3 Distribution of variables with missing data in all populations Table S4 Characteristics of the population that developed stroke and those without, based on data from 2011 Table S5 Characteristics of the population that developed stroke and those without, based on data from 2011 Table S6 Association between VAII and the risk of stroke according to gender after excluding missing data Table S7 Association between VAII and the risk of stroke according to age after excluding missing data Table S8 Association between VAII and stroke risk according to blood pressure regulation status after multiple imputation Table S9 Association between VAII and the risk of stroke according to gender after multiple imputation Table S10 Association between VAII and the risk of stroke according to age after multiple imputation Table S11 Association between VAII and stroke risk by blood pressure status full‐cohort multiple imputation Table S12 Association between VAII and stroke risk by blood pressure status after excluding stroke events within the first two years of follow‐up Table S13 Association of VAII with fall injury across blood pressure status after full‐cohort multiple imputation Figure S1 Flowchart of the study population. Figure S2 Flowchart of the study population following full multiple imputation with 20 datasets in the original cohort. Figure S3 E‐value plot evaluating the risk ratios of VAII in relation to stroke risk. Adjusted for sex, age, residence, marital status, education level, smoking, drinking, sleep time, DM, cancer, pulmonary disease, heart disease, dyslipidemia, liver disease, and kidney disease. [file BRB3-15-e71147-s001.docx]

| Table S1. Distribution of variables with missing data | | |
| --- | --- | --- |
| **Variables** | **Number of Missing** | **Missing proportion** |
| Cancer | 30 | 0.34% |
| Diabetes mellitus | 69 | 0.78% |
| Smoking | 1 | 0.01% |
| Drinking | 4 | 0.05% |
| Sleep time | 120 | 1.35% |
| Dyslipidemia | 147 | 1.65% |
| Glucose | 9 | 0.10% |
| Heart disease | 27 | 0.3% |
| Kidney disease | 34 | 0.38% |
| Liver disease | 45 | 0.51% |
| Pulmonary disease | 26 | 0.29% |

| Table S2. Assessment of collinearity among independent variables in the final regression model | | | | |
| --- | --- | --- | --- | --- |
| **Variables** | **GVIF** | **Df** | **Adjusted GVIF** | **Collinearity** |
| Age | 1.25 | 1 | 1.118 | 0 |
| Sex | 2.37 | 1 | 1.539 | 0 |
| Education level | 1.345 | 3 | 1.051 | 0 |
| Residence | 1.086 | 1 | 1.042 | 0 |
| Marital status | 1.132 | 1 | 1.064 | 0 |
| Smoking | 2.03 | 2 | 1.194 | 0 |
| Drinking | 1.503 | 2 | 1.107 | 0 |
| Sleep time | 1.026 | 1 | 1.013 | 0 |
| Diabetes mellitus | 1.097 | 1 | 1.047 | 0 |
| Cancer | 1.012 | 1 | 1.006 | 0 |
| Pulmonary disease | 1.031 | 1 | 1.015 | 0 |
| Heart disease | 1.126 | 1 | 1.061 | 0 |
| Dyslipidemia | 1.16 | 1 | 1.077 | 0 |
| Liver disease | 1.02 | 1 | 1.01 | 0 |
| Kidney disease | 1.033 | 1 | 1.016 | 0 |

| Table S3. Distribution of variables with missing data in all population | | |
| --- | --- | --- |
| **Variables** | **Number of Missing** | **Missing proportion (%)** |
| Age | 40 | 0.25 |
| BMI | 3347 | 20.945 |
| Cancer | 62 | 0.388 |
| Diabetes mellitus | 124 | 0.776 |
| Drinking | 24 | 0.15 |
| Education level | 21 | 0.131 |
| HDL-C | 5117 | 32.021 |
| Heart disease | 69 | 0.432 |
| Kidney disease | 81 | 0.507 |
| LDL-C | 5136 | 32.14 |
| Liver disease | 102 | 0.638 |
| Pulmonary disease | 52 | 0.325 |
| Waist circumference | 3243 | 20.294 |
| Sleep time | 1233 | 7.716 |
| Smoking | 14 | 0.088 |
| TC | 5124 | 32.065 |
| TG | 5123 | 32.059 |

| Table S4. Characteristics of the population that developed stroke and those without, based on data from 2011 | | | |
| --- | --- | --- | --- |
| Characteristics | No occurrence of stroke (n = 8,056) | Occurrence of stroke (n = 827) | P value |
| Age, years | 59.11 ± 9.26 | 61.35 ± 8.45 | < 0.001 |
| Sex (Female), n (%) | 4340 (53.87) | 434 (52.48) | 0.444 |
| Residence (Urban), n (%) | 2793 (34.67) | 287 (34.7) | 0.984 |
| Education level, n (%) |  |  | 0.238 |
| No formal education | 3871 (48.09) | 403 (48.73) |  |
| Primary school | 1751 (21.75) | 199 (24.06) |  |
| Middle school | 1619 (20.11) | 153 (18.5) |  |
| High school or above | 809 (10.05) | 72 (8.71) |  |
| Current married, n (%) | 7098 (88.11) | 709 (85.73) | 0.046 |
| SBP, mmHg | 128.59 ± 21.00 | 136.90 ± 23.46 | < 0.001 |
| DBP, mmHg | 74.97 ± 12.07 | 78.63 ± 12.73 | < 0.001 |
| Waist circumference, cm | 83.98 ± 12.45 | 87.43 ± 13.12 | < 0.001 |
| BMI, kg/m2 | 23.05 (20.78, 25.64) | 24.06 (21.70, 26.82) | < 0.001 |
| Sleep time, hour | 6.37 ± 1.87 | 6.17 ± 1.95 | 0.004 |
| Smoking |  |  | 0.004 |
| Never, n (%) | 4948 (61.43) | 474 (57.32) |  |
| Former, n (%) | 684 (8.49) | 97 (11.73) |  |
| Current, n (%) | 2423 (30.08) | 256 (30.96) |  |
| Drinking |  |  | 0.013 |
| Never, n (%) | 4767 (59.2) | 470 (56.9) |  |
| Former, n (%) | 631 (7.84) | 89 (10.77) |  |
| Current, n (%) | 2655 (32.97) | 267 (32.32) |  |
| **Comorbidities** |  |  |  |
| DM, n (%) | 449 (5.62) | 75 (9.11) | < 0.001 |
| Cancer, n (%) | 70 (0.87) | 5 (0.61) | 0.427 |
| Pulmonary disease, n (%) | 772 (9.61) | 83 (10.04) | 0.695 |
| Heart disease, n (%) | 866 (10.78) | 163 (19.76) | < 0.001 |
| Dyslipidemia, n (%) | 687 (8.68) | 144 (17.58) | < 0.001 |
| Liver disease, n (%) | 259 (3.23) | 33 (4) | 0.24 |
| Kidney disease, n (%) | 443 (5.52) | 59 (7.15) | 0.054 |
| Digestive system disease, n (%) | 1858 (23.1) | 182 (22.01) | 0.477 |
| **Laboratory parameters** |  |  |  |
| BUN, mg/dl | 15.75 ± 4.50 | 15.68 ± 4.40 | 0.673 |
| Scr, mg/dl | 0.78 ± 0.23 | 0.80 ± 0.19 | 0.002 |
| Glucose, mg/dl | 6.09 ± 1.97 | 6.45 ± 2.41 | < 0.001 |
| TC, mmol/L | 5.01 ± 1.00 | 5.11 ± 1.00 | 0.007 |
| TG, mmol/L | 1.18 (0.84, 1.72) | 1.33 (0.96, 1.90) | < 0.001 |
| HDL-C, mmol/L | 1.14 ± 0.34 | 1.08 ± 0.33 | < 0.001 |
| LDL-C, mmol/L | 3.01 ± 0.90 | 3.10 ± 0.93 | 0.009 |
| hs-CRP, mg/L | 0.98 (0.54, 2.09) | 1.33 (0.68, 2.66) | < 0.001 |
| VAI | 1.70 (1.01, 3.00) | 2.07 (1.26, 3.56) | < 0.001 |
| VAII | 1.83 (0.72, 5.16) | 2.98 (1.20, 7.78) | < 0.001 |

SBP, Systolic blood pressure; DBP, Diastolic blood pressure; BMI, Body mass index; DM, Diabetes mellitus; BUN, Blood urea nitrogen; Scr, Serum creatinine; TC, Total cholesterol; TG, Triglycerides; HDL-C, High-density lipoprotein cholesterol; LDL-C, Low-density lipoprotein cholesterol; hs-CRP, High-sensitivity C-reactive protein; VAI, Visceral adiposity index; VAII, Visceral adiposity and inflammation index.

| Table S5.  Association between VAII and stroke risk according to blood pressure regulation status after excluding missing data | | | | | | | | | | |
| --- | --- | --- | --- | --- | --- | --- | --- | --- | --- | --- |
| Categories | Characteristic | Event, (n%) | Model 1 | |  | Model 2 | |  | Model 3 | |
|  |  |  | HR (95% CI) | P value |  | HR (95% CI) | P value |  | HR (95% CI) | P value |
| Total | VAII  (per 1 SD) | 800 (9.4) | 1.055  (1.023, 1.089) | <0.001 |  | 1.055  (1.022, 1.089) | 0.001 |  | 1.048  (1.009, 1.089) | 0.017 |
|  | VAII |  |  |  |  |  |  |  |  |  |
|  | Q1 | 124 (5.8) | 1(Ref) |  |  | 1(Ref) |  |  | 1(Ref) |  |
|  | Q2 | 177 (8.3) | 1.482  (1.178, 1.864) | <0.001 |  | 1.449  (1.151, 1.825) | 0.002 |  | 1.43  (1.135, 1.801) | 0.002 |
|  | Q3 | 227 (10.7) | 1.941  (1.559, 2.415) | <0.001 |  | 1.893  (1.518, 2.362) | <0.001 |  | 1.806  (1.446, 2.254) | <0.001 |
|  | Q4 | 272 (12.8) | 2.432  (1.967, 3.008) | <0.001 |  | 2.373  (1.911, 2.945) | <0.001 |  | 2.172  (1.745, 2.703) | <0.001 |
|  | P for trend |  | 1.33  (1.248, 1.417) | <0.001 |  | 1.321  (1.237, 1.409) | <0.001 |  | 1.28  (1.199, 1.368) | <0.001 |
| Normal BP | VAII  (per 1 SD) | 106 (5) | 1.192  (0.967, 1.469) | 0.01 |  | 1.213  (0.977, 1.506) | 0.08 |  | 1.189  (0.949, 1.49) | 0.132 |
|  | VAII | 20 (3.7) | 1(Ref) |  |  | 1(Ref) |  |  | 1(Ref) |  |
|  | Q1 | 26 (4.8) | 1.306  (0.729, 2.34) | 0.369 |  | 1.253  (0.698, 2.251) | 0.450 |  | 1.338  (0.742, 2.412) | 0.333 |
|  | Q2 | 33 (6.1) | 1.669  (0.958, 2.909) | 0.071 |  | 1.657  (0.946, 2.9) | 0.077 |  | 1.668  (0.95, 2.928) | 0.075 |
|  | Q3 | 27 (5.2) | 1.506  (0.845, 2.685) | 0.165 |  | 1.446  (0.804, 2.599) | 0.218 |  | 1.494  (0.827, 2.7) | 0.184 |
|  | Q4 |  | 1.152  (0.969, 1.369) | 0.109 |  | 1.142  (0.959, 1.361) | 0.136 |  | 1.146  (0.96, 1.367) | 0.131 |
|  | P for trend |  |  |  |  |  |  |  |  |  |
|  | VAII  (per 1 SD) | 221 (7.3) | 1.027  (0.956, 1.103) | 0.471 |  | 1.022  (0.952, 1.097) | 0.553 |  | 1.027  (0.956, 1.104) | 0.462 |
|  | VAII |  |  |  |  |  |  |  |  |  |
|  | Q1 | 41 (5.4) | 1(Ref) |  |  | 1(Ref) |  |  | 1(Ref) |  |
|  | Q2 | 44 (5.9) | 1.121  (0.733, 1.716) | 0.598 |  | 1.115  (0.727, 1.71) | 0.617 |  | 1.109  (0.722, 1.703) | 0.637 |
|  | Q3 | 59 (7.8) | 1.501  (1.008, 2.236) | 0.046 |  | 1.527  (1.019, 2.287) | 0.040 |  | 1.518  (1.012, 2.277) | 0.044 |
|  | Q4 | 77 (10) | 2.035  (1.393, 2.973) | <0.001 |  | 2.109 (  1.431, 3.11) | <0.001 |  | 2.034  (1.376, 3.007) | <0.001 |
|  | P for trend |  | 1.284  (1.138, 1.448) | <0.001 |  | 1.302  (1.151, 1.474) | <0.001 |  | 1.286  (1.136, 1.457) | <0.001 |
| Hypertension | VAII  (per 1 SD) | 473 (14.1) | 1.062  (1, 1.128) | 0.051 |  | 1.059  (0.999, 1.124) | 0.055 |  | 1.041  (0.975, 1.111) | 0.227 |
|  | VAII |  |  |  |  |  |  |  |  |  |
|  | Q1 | 80 (9.5) | 1(Ref) |  |  | 1(Ref) |  |  | 1(Ref) |  |
|  | Q2 | 111 (13.2) | 1.433  (1.075, 1.91) | 0.014 |  | 1.414  (1.059, 1.887) | 0.019 |  | 1.383  (1.036, 1.847) | 0.028 |
|  | Q3 | 147 (17.5) | 1.883  (1.434, 2.473) | <0.001 |  | 1.912  (1.451, 2.519) | <0.001 |  | 1.78  (1.348, 2.351) | <0.001 |
|  | Q4 | 135 (16.1) | 1.824  (1.383, 2.405) | <0.001 |  | 1.873  (1.413, 2.483) | <0.001 |  | 1.701  (1.277, 2.265) | <0.001 |
|  | P for trend |  | 1.215  (1.119, 1.319) | <0.001 |  | 1.229  (1.13, 1.336) | <0.001 |  | 1.188  (1.091, 1.295) | <0.001 |
| Model 1:unadjusted for any covariates Model 2: adjusted for gender, sex, residence, marital status, education level, smoking status, and drinking status Model 3: adjusted for gender, sex, residence, marital status, education level, smoking, drinking, sleep time, DM, cancer, pulmonary disease, heart disease, dyslipidemia, liver disease, kidney disease.  BP, Blood pressure; VAII, Visceral adiposity and inflammation index; SD, Standard deviation. | | | | | | | | | | |
|  |  |  |  |  |  |  |  |  |  |  |
|  |  |  |  |  |  |  |  |  |  |  |

| Table S6.  Association between VAII and the risk of stroke according to gender after excluding missing data | | | | | | | | | | |
| --- | --- | --- | --- | --- | --- | --- | --- | --- | --- | --- |
| Sex | Characteristic | Event, (n%) | Model 1 | |  | Model 2 | |  | Model 3 | |
|  |  |  | HR (95% CI) | P value |  | HR (95% CI) | P value |  | HR (95% CI) | P value |
| Male | VAII  (per 1 SD) | 382 (9.6) | 1.039  (0.991, 1.089) | 0.113 |  | 1.04  (0.991, 1.091) | 0.108 |  | 1.037  (0.984, 1.093) | 0.17 |
|  | VAII |  |  |  |  |  |  |  |  |  |
|  | Q1 | 62 (6.2) | 1(Ref) |  |  | 1(Ref) |  |  | 1(Ref) |  |
|  | Q2 | 76 (7.7) | 1.268  (0.907, 1.774) | 0.165 |  | 1.238  (0.885, 1.732) | 0.213 |  | 1.205  (0.861, 1.686) | 0.278 |
|  | Q3 | 111 (11.2) | 1.896  (1.389, 2.587) | <0.001 |  | 1.812  (1.325, 2.479) | <0.001 |  | 1.729  (1.263, 2.368) | <0.001 |
|  | Q4 | 133 (13.4) | 2.406  (1.78, 3.253) | <0.001 |  | 2.349  (1.734, 3.183) | <0.001 |  | 2.108  (1.55, 2.868) | <0.001 |
|  | P for trend |  | 1.354  (1.234, 1.485) | <0.001 |  | 1.344  (1.224, 1.477) | <0.001 |  | 1.296  (1.179, 1.426) | <0.001 |
| Female | VAII  (per 1 SD) | 418 (9.2) | 1.104 (1.044, 1.169) | <0.001 |  | 1.096 (1.034, 1.162) | 0.002 |  | 1.068 (0.999, 1.143) | 0.054 |
|  | VAII |  |  |  |  |  |  |  |  |  |
|  | Q1 | 61 (5.4) | 1(Ref) |  |  | 1(Ref) |  |  | 1(Ref) |  |
|  | Q2 | 95 (8.3) | 1.62  (1.175, 2.235) | 0.003 |  | 1.516  (1.098, 2.093) | 0.012 |  | 1.501  (1.087, 2.072) | 0.014 |
|  | Q3 | 120 (10.5) | 2.102  (1.545, 2.861) | <0.001 |  | 1.889  (1.386, 2.576) | <0.001 |  | 1.825  (1.338, 2.49) | <0.001 |
|  | Q4 | 142 (12.5) | 2.614  (1.937, 3.529) | <0.001 |  | 2.321  (1.714, 3.145) | <0.001 |  | 2.154  (1.584, 2.929) | <0.001 |
|  | P for trend |  | 1.349  (1.235, 1.474) | <0.001 |  | 1.3  (1.188, 1.423) | <0.001 |  | 1.267  (1.156, 1.388) | <0.001 |

Model 1:unadjusted for any covariates
Model 2: adjusted for sex, age, residence, marital status, education level, smoking status, and drinking status
Model 3: adjusted for sex, age, residence, marital status, education level, smoking, drinking, sleep time, DM, cancer, pulmonary disease, heart disease, dyslipidemia, liver disease, kidney disease.
VAII, Visceral adiposity and inflammation index; SD, Standard deviation.

| Table S7.  Association between VAII and the risk of stroke according to age after excluding missing data | | | | | | | | | | | |
| --- | --- | --- | --- | --- | --- | --- | --- | --- | --- | --- | --- |
| Age | Characteristic | Event, (n%) | Model 1 | |  | Model 12 | |  | Model 3 | | |
|  |  |  | HR (95% CI) | P value |  | HR (95%CI) | P value |  | HR (95% CI) | P value | |
| 40-60 | VAII  (per 1 SD) | 349 (7.4) | 1.054  (1.012, 1.098) | 0.011 |  | 1.054  (1.012, 1.098) | 0.011 |  | 1.042  (0.992, 1.094) | 0.102 | |
|  | VAII |  |  |  |  |  |  |  |  |  | |
|  | Q1 | 50 (4.3) | 1(Ref) |  |  | 1(Ref) |  |  | 1(Ref) |  | |
|  | Q2 | 76 (6.5) | 1.535  (1.074, 2.193) | 0.019 |  | 1.543  (1.079, 2.207) | 0.018 |  | 1.523  (1.065, 2.179) | 0.021 | |
|  | Q3 | 96 (8.2) | 1.993  (1.416, 2.805) | <0.001 |  | 1.988  (1.409, 2.805) | <0.001 |  | 1.854  (1.311, 2.62) | <0.001 | |
|  | Q4 | 127 (10.8) | 2.693  (1.941, 3.736) | <0.001 |  | 2.689  (1.931, 3.746) | <0.001 |  | 2.353  (1.68, 3.296) | <0.001 | |
|  | P for trend |  | 1.371  (1.244, 1.511) | <0.001 |  | 1.369  (1.24, 1.511) | <0.001 |  | 1.305  (1.18, 1.443) | <0.001 | |
| >= 60 | VAII  (per 1 SD) | 451 (11.8) | 1.07  (1.002, 1.143) | 0.043 |  | 1.067  (0.996, 1.143) | 0.065 |  | 1.057  (0.98, 1.14) | 0.149 |  |
|  | VAII |  |  |  |  |  |  |  |  |  | |
|  | Q1 | 82 (8.6) | 1(Ref) |  |  | 1(Ref) |  |  | 1(Ref) |  | |
|  | Q2 | 96 (10) | 1.215  (0.905, 1.632) | 0.195 |  | 1.219  (0.907, 1.64) | 0.189 |  | 1.219  (0.907, 1.64) | 0.19 | |
|  | Q3 | 133 (13.9) | 1.724  (1.309, 2.27) | <0.001 |  | 1.71  (1.294, 2.261) | <0.001 |  | 1.661  (1.256, 2.198) | <0.001 | |
|  | Q4 | 140 (14.6) | 1.93  (1.47, 2.535) | <0.001 |  | 1.929  (1.457, 2.553) | <0.001 |  | 1.822  (1.373, 2.419) | <0.001 | |
|  | P for trend |  | 1.258  (1.156, 1.368) | <0.001 |  | 1.256  (1.152, 1.37) | <0.001 |  | 1.23  (1.127, 1.343) | <0.001 | |

Model 1:unadjusted for any covariates
Model 2: adjusted for sex, age, residence, marital status, education level, smoking status, and drinking status
Model 3: adjusted for sex, age, residence, marital status, education level, smoking, drinking, sleep time, DM, cancer, pulmonary disease, heart disease, dyslipidemia, liver disease, kidney disease.
VAII, Visceral adiposity and inflammation index; SD, Standard deviation.

| Table S8.  Association between VAII and stroke risk according to blood pressure regulation status after multiple imputation | | | | | | | | | | |
| --- | --- | --- | --- | --- | --- | --- | --- | --- | --- | --- |
| Categories | Characteristic | Event, (n%) | Model 1 | |  | Model 2 | |  | Model 3 | |
|  |  |  | HR (95% CI) | P value |  | HR (95% CI) | P value |  | HR (95% CI) | P value |
| Total | VAII  (per 1 SD) | 800 (9.4 | 1.053  (1.019, 1.087) | 0.002 |  | 1.052  (1.018, 1.087) | 0.002 |  | 1.044  (1.004, 1.085) | 0.029 |
|  | VAII |  |  |  |  |  |  |  |  |  |
|  | Q1 | 124 (5.8) | 1(Ref) |  |  | 1(Ref) |  |  | 1(Ref) |  |
|  | Q2 | 177 (8.3) | 1.482  (1.178, 1.864) | <0.001 |  | 1.449  (1.151, 1.825) | 0.002 |  | 1.43  (1.135, 1.801) | 0.002 |
|  | Q3 | 227 (10.7) | 1.941  (1.559, 2.415) | <0.001 |  | 1.893  (1.518, 2.362) | <0.001 |  | 1.806  (1.446, 2.254) | <0.001 |
|  | Q4 | 272 (12.8) | 2.432  (1.967, 3.008) | <0.001 |  | 2.373  (1.911, 2.945) | <0.001 |  | 2.172  (1.745, 2.703) | <0.001 |
|  | P for trend |  | 1.33  (1.248, 1.417) | <0.001 |  | 1.321  (1.237, 1.409) | <0.001 |  | 1.28  (1.199, 1.368) | <0.001 |
| Normal BP | VAII  (per 1 SD) | 106 (5) | 1.053 (0.952, 1.164) | 0.318 |  | 1.062 (0.957, 1.178) | 0.256 |  | 1.073 (0.953, 1.207) | 0.2433 |
|  | VAII | 20 (3.7) | 1(Ref) |  |  | 1(Ref) |  |  | 1(Ref) |  |
|  | Q1 | 26 (4.8) | 1.306  (0.729, 2.34) | 0.369 |  | 1.253  (0.698, 2.251) | 0.450 |  | 1.338  (0.742, 2.412) | 0.333 |
|  | Q2 | 33 (6.1) | 1.669  (0.958, 2.909) | 0.071 |  | 1.657  (0.946, 2.9) | 0.077 |  | 1.668  (0.95, 2.928) | 0.075 |
|  | Q3 | 27 (5.2) | 1.506  (0.845, 2.685) | 0.165 |  | 1.446  (0.804, 2.599) | 0.218 |  | 1.494  (0.827, 2.7) | 0.184 |
|  | Q4 |  | 1.152  (0.969, 1.369) | 0.109 |  | 1.142  (0.959, 1.361) | 0.136 |  | 1.146  (0.96, 1.367) | 0.131 |
|  | P for trend |  |  |  |  |  |  |  |  |  |
| Elevated BP | VAII  (per 1 SD) | 221 (7.3) | 1.026  (0.948, 1.111) | 0.519 |  | 1.02  (0.942, 1.105) | 0.625 |  | 1.028  (0.95, 1.111) | 0.492 |
|  | VAII |  |  |  |  |  |  |  |  |  |
|  | Q1 | 41 (5.4) | 1(Ref) |  |  | 1(Ref) |  |  | 1(Ref) |  |
|  | Q2 | 44 (5.9) | 1.121  (0.733, 1.716) | 0.598 |  | 1.115  (0.727, 1.71) | 0.617 |  | 1.109  (0.722, 1.703) | 0.637 |
|  | Q3 | 59 (7.8) | 1.501  (1.008, 2.236) | 0.046 |  | 1.527  (1.019, 2.287) | 0.040 |  | 1.518  (1.012, 2.277) | 0.044 |
|  | Q4 | 77 (10) | 2.035  (1.393, 2.973) | <0.001 |  | 2.109 (  1.431, 3.11) | <0.001 |  | 2.034  (1.376, 3.007) | <0.001 |
|  | P for trend |  | 1.284  (1.138, 1.448) | <0.001 |  | 1.302  (1.151, 1.474) | <0.001 |  | 1.286  (1.136, 1.457) | <0.001 |
| Hypertension | VAII  (per 1 SD) | 473 (14.1) | 1.067  (1.001, 1.137) | 0.048 |  | 1.065  (1, 1.134) | 0.048 |  | 1.045  (0.976, 1.12) | 0.208 |
|  | VAII |  |  |  |  |  |  |  |  |  |
|  | Q1 | 80 (9.5) | 1(Ref) |  |  | 1(Ref) |  |  | 1(Ref) |  |
|  | Q2 | 111 (13.2) | 1.433  (1.075, 1.91) | 0.014 |  | 1.414  (1.059, 1.887) | 0.019 |  | 1.383  (1.036, 1.847) | 0.028 |
|  | Q3 | 147 (17.5) | 1.883  (1.434, 2.473) | <0.001 |  | 1.912  (1.451, 2.519) | <0.001 |  | 1.78  (1.348, 2.351) | <0.001 |
|  | Q4 | 135 (16.1) | 1.824  (1.383, 2.405) | <0.001 |  | 1.873  (1.413, 2.483) | <0.001 |  | 1.701  (1.277, 2.265) | <0.001 |
|  | P for trend |  | 1.215  (1.119, 1.319) | <0.001 |  | 1.229  (1.13, 1.336) | <0.001 |  | 1.188  (1.091, 1.295) | <0.001 |
| Model 1:unadjusted for any covariates Model 2: adjusted for sex, age, residence, marital status, education level, smoking status, and drinking status Model 3: adjusted for sex, age, residence, marital status, education level, smoking, drinking, sleep time, DM, cancer, pulmonary disease, heart disease, dyslipidemia, liver disease, kidney disease.  BP, Blood pressure; VAII, Visceral adiposity and inflammation index; SD, Standard deviation. | | | | | | | | | | |
|  |  |  |  |  |  |  |  |  |  |  |
|  |  |  |  |  |  |  |  |  |  |  |

| Table S9.  Association between VAII and the risk of stroke according to gender after multiple imputation | | | | | | | | | | |
| --- | --- | --- | --- | --- | --- | --- | --- | --- | --- | --- |
| Sex | Characteristic | Event, (n%) | Model 1 | |  | Model 2 | |  | Model 3 | |
|  |  |  | HR (95% CI) | P value |  | HR (95% CI) | P value |  | HR (95% CI) | P value |
| Male | VAII  (per 1 SD) | 393 (9.6) | 1.038  (0.991, 1.087) | 0.118 |  | 1.039  (0.991, 1.089) | 0.114 |  | 1.037  (0.985, 1.091) | 0.17 |
|  | VAII |  |  |  |  |  |  |  |  |  |
|  | Q1 | 64 (6.2) | 1(Ref) |  |  | 1(Ref) |  |  | 1(Ref) |  |
|  | Q2 | 79 (7.7) | 1.281  (0.921, 1.782) | 0.141 |  | 1.248  (0.897, 1.736) | 0.188 |  | 1.199  (0.857, 1.678) | 0.290 |
|  | Q3 | 115 (11.2) | 1.898  (1.398, 2.576) | <0.001 |  | 1.806  (1.326, 2.458) | <0.001 |  | 1.708  (1.247, 2.34) | <0.001 |
|  | Q4 | 135 (13.1) | 2.361  (1.754, 3.179) | <0.001 |  | 2.313  (1.714, 3.121) | <0.001 |  | 2.105  (1.548, 2.863) | <0.001 |
|  | P for trend |  | 1.343  (1.226, 1.471) | <0.001 |  | 1.335  (1.217, 1.465) | <0.001 |  | 1.296  (1.178, 1.425) | <0.001 |
| Female | VAII  (per 1 SD) | 434 (9.1) | 1.085  (1.028, 1.146) | 0.003 |  | 1.078  (1.02, 1.14) | 0.008 |  | 1.054  (0.989, 1.123) | 0.105 |
|  | VAII |  |  |  |  |  |  |  |  |  |
|  | Q1 | 64 (5.4) | 1(Ref) |  |  | 1(Ref) |  |  | 1(Ref) |  |
|  | Q2 | 101 (8.5) | 1.644  (1.202, 2.248) | 0.002 |  | 1.536  (1.122, 2.103) | 0.007 |  | 1.529  (1.117, 2.094) | 0.008 |
|  | Q3 | 120 (10.1) | 2.004  (1.48, 2.714) | <0.001 |  | 1.804  (1.329, 2.447) | <0.001 |  | 1.746  (1.286, 2.371) | <0.001 |
|  | Q4 | 149 (12.5) | 2.619  (1.954, 3.511) | <0.001 |  | 2.32  (1.725, 3.121) | <0.001 |  | 2.159  (1.599, 2.914) | <0.001 |
|  | P for trend |  | 1.344  (1.233, 1.466) | <0.001 |  | 1.294  (1.185, 1.414) | <0.001 |  | 1.261  (1.153, 1.379) | <0.001 |

Model 1:unadjusted for any covariates
Model 2: adjusted for sex, age, residence, marital status, education level, smoking status, and drinking status
Model 3: adjusted for sex, age, residence, marital status, education level, smoking, drinking, sleep time, DM, cancer, pulmonary disease, heart disease, dyslipidemia, liver disease, kidney disease.
VAII, Visceral adiposity and inflammation index; SD, Standard deviation.

| Table S10.  Association between VAII and the risk of stroke according to age after multiple imputation | | | | | | | | | | |
| --- | --- | --- | --- | --- | --- | --- | --- | --- | --- | --- |
| Age | Characteristic | Event, (n%) | Model 1 | |  | Model 12 | |  | Model 3 | |
|  |  |  | HR (95% CI) | P value |  | HR (95% CI) | P value |  | HR (95% CI) | P value |
| 40-60 | VAII  (per 1 SD) | 358 (7.4) | 1.052  (1.006, 1.101) | 0.026 |  | 1.052  (1.006, 1.101) | 0.026 |  | 1.04  (0.986, 1.097) | 0.153 |
|  | VAII |  |  |  |  |  |  |  |  |  |
|  | Q1 | 51 (4.2) | 1(Ref) |  |  | 1(Ref) |  |  | 1(Ref) |  |
|  | Q2 | 79 (6.5) | 1.571  (1.105, 2.235) | 0.012 |  | 1.596  (1.121, 2.271) | 0.010 |  | 1.577  (1.108, 2.246) | 0.012 |
|  | Q3 | 97 (8) | 1.977  (1.409, 2.775) | <0.001 |  | 2.029  (1.442, 2.855) | <0.001 |  | 1.873  (1.329, 2.641) | <0.001 |
|  | Q4 | 131 (10.8) | 2.734  (1.978, 3.779) | <0.001 |  | 2.815  (2.03, 3.903) | <0.001 |  | 2.422 (1.736, 3.38) | <0.001 |
|  | P for trend |  | 1.373  (1.247, 1.512) | <0.001 |  | 1.385  (1.257, 1.527) | <0.001 |  | 1.312  (1.188, 1.448) | <0.001 |
| >= 60 | VAII  (per 1 SD) | 469 (11.7) | 1.068  (1.006, 1.134) | 0.03 |  | 1.065  (1.001, 1.134) | 0.046 |  | 1.057  (0.987, 1.131) | 0.112 |
|  | VAII |  |  |  |  |  |  |  |  |  |
|  | Q1 | 86 (8.6) | 1(Ref) |  |  | 1(Ref) |  |  | 1(Ref) |  |
|  | Q2 | 102 (10.2) | 1.245  (0.934, 1.659) | 0.134 |  | 1.253  (0.939, 1.672) | 0.125 |  | 1.223  (0.909, 1.645) | 0.183 |
|  | Q3 | 135 (13.4) | 1.673  (1.276, 2.192) | <0.001 |  | 1.652  (1.255, 2.173) | <0.001 |  | 1.65  (1.247, 2.184) | <0.001 |
|  | Q4 | 146 (14.5) | 1.928  (1.477, 2.516) | <0.001 |  | 1.924  (1.463, 2.531) | <0.001 |  | 1.827  (1.377, 2.425) | <0.001 |
|  | P for trend |  | 1.251  (1.152, 1.358) | <0.001 |  | 1.248  (1.146, 1.358) | <0.001 |  | 1.23  (1.127, 1.343) | <0.001 |

Model 1:unadjusted for any covariates
Model 2: adjusted for gender, age, residence, marital status, education level, smoking status, and drinking status
Model 3: adjusted for gender, age, residence, marital status, education level, smoking, drinking, sleep time, DM, cancer, pulmonary disease, heart disease, dyslipidemia, liver disease, kidney disease.
VAII, Visceral adiposity and inflammation index; SD, Standard deviation.

| Table S11.  Association between VAII and stroke risk by blood pressure status full-cohort multiple imputation | | | | | | | | | | |
| --- | --- | --- | --- | --- | --- | --- | --- | --- | --- | --- |
| Categories | Characteristic | Event, (n%) | Model 1 | |  | Model 2 | |  | Model 3 | |
|  |  |  | HR (95% CI) | P value |  | HR (95% CI) | P value |  | HR (95% CI) | P value |
| Total | VAII  (per 1 SD) | 1324 (8.5) | 1.06  (1.036, 1.085) | <0.001 |  | 1.06  (1.035, 1.085) | <0.001 |  | 1.051  (1.02, 1.082) | <0.001 |
|  | VAII |  |  |  |  |  |  |  |  |  |
|  | Q1 | 230 (5.9) | 1(Ref) |  |  | 1(Ref) |  |  | 1(Ref) |  |
|  | Q2 | 282 (7.3) | 1.267  (1.065, 1.508) | 0.008 |  | 1.279  (1.074, 1.522) | 0.006 |  | 1.248  (1.048, 1.487) | 0.013 |
|  | Q3 | 356 (9.2) | 1.638  (1.388, 1.934) | <0.001 |  | 1.647  (1.393, 1.946) | <0.001 |  | 1.54  (1.302, 1.821) | <0.001 |
|  | Q4 | 456 (11.8) | 2.214  (1.889, 2.594) | <0.001 |  | 2.233  (1.902, 2.622) | <0.001 |  | 1.934  (1.641, 2.279) | <0.001 |
|  | P for trend |  | 1.306  (1.243, 1.372) | <0.001 |  | 1.309  (1.245, 1.376) | <0.001 |  | 1.245  (1.183, 1.31) | <0.001 |
| Normal BP | VAII  (per 1 SD) | 256 (4.9) | 1.09  (1.039, 1.143) | <0.001 |  | 1.091  (1.039, 1.146) | <0.001 |  | 1.096  (1.036, 1.158) | 0.001 |
|  | VAII |  |  |  |  |  |  |  |  |  |
|  | Q1 | 47 (3.6) | 1(Ref) |  |  | 1(Ref) |  |  | 1(Ref) |  |
|  | Q2 | 57 (4.4) | 1.247  (0.848, 1.835) | 0.262 |  | 1.255  (0.852, 1.848) | 0.25 |  | 1.234  (0.836, 1.819) | 0.23 |
|  | Q3 | 64 (4.9) | 1.456  (0.999, 2.121) | 0.051 |  | 1.473  (1.009, 2.151) | 0.045 |  | 1.421  (0.972, 2.078) | 0.07 |
|  | Q4 | 88 (6.8) | 2.128  (1.494, 3.033) | <0.001 |  | 2.125  (1.486, 3.038) | <0.001 |  | 1.992  (1.384, 2.866) | 2e-04 |
|  | P for trend |  | 1.283  (1.147, 1.435) | <0.001 |  | 1.282  (1.145, 1.435) | <0.001 |  | 1.254  (1.118, 1.406) | 1e-04 |
| Elevated BP | VAII  (per 1 SD) | 238 (6.5) | 1.025  (0.951, 1.106) | 0.516 |  | 1.022  (0.944, 1.107) | 0.595 |  | 1.024  (0.94, 1.115) | 0.59 |
|  | VAII |  |  |  |  |  |  |  |  |  |
|  | Q1 | 49 (5.4) | 1(Ref) |  |  | 1(Ref) |  |  | 1(Ref) |  |
|  | Q2 | 51 (5.6) | 1.087  (0.735, 1.609) | 0.676 |  | 1.066  (0.719, 1.581) | 0.75 |  | 1.037  (0.699, 1.54) | 0.855 |
|  | Q3 | 58 (6.4) | 1.214  (0.83, 1.776) | 0.317 |  | 1.218  (0.83, 1.788) | 0.314 |  | 1.173  (0.798, 1.723) | 0.417 |
|  | Q4 | 80 (8.8) | 1.755  (1.23, 2.505) | 0.002 |  | 1.722  (1.199, 2.473) | 0.003 |  | 1.579  (1.093, 2.28) | 0.015 |
|  | P for trend |  | 1.209  (1.077, 1.356) | 0.001 |  | 1.205  (1.072, 1.354) | 0.002 |  | 1.171  (1.04, 1.318) | 0.009 |
| Hypertension | VAII  (per 1 SD) | 830 (12.5) | 1.071  (1.022, 1.123) | 0.004 |  | 1.072  (1.023, 1.124) | 0.003 |  | 1.05  (0.997, 1.107) | 0.066 |
|  | VAII |  |  |  |  |  |  |  |  |  |
|  | Q1 | 149 (8.9) | 1(Ref) |  |  | 1(Ref) |  |  | 1(Ref) |  |
|  | Q2 | 203 (12.2) | 1.401  (1.134, 1.731) | 0.002 |  | 1.423  (1.151, 1.759) | 0.001 |  | 1.393  (1.127, 1.723) | 0.002 |
|  | Q3 | 229 (13.7) | 1.594  (1.297, 1.959) | <0.001 |  | 1.631  (1.325, 2.009) | <0.001 |  | 1.55  (1.256, 1.912) | <0.001 |
|  | Q4 | 249 (14.9) | 1.837  (1.499, 2.251) | <0.001 |  | 1.907  (1.552, 2.343) | <0.001 |  | 1.737  (1.408, 2.144) | <0.001 |
|  | P for trend |  | 1.207  (1.135, 1.284) | <0.001 |  | 1.222  (1.147, 1.301) | <0.001 |  | 1.184  (1.11, 1.263) | <0.001 |
| Model 1:unadjusted for any covariates Model 2: adjusted for gender, age, residence, marital status, education level, smoking status, and drinking status Model 3: adjusted for gender, age, residence, marital status, education level, smoking, drinking, sleep time, DM, cancer, pulmonary disease, heart disease, dyslipidemia, liver disease, kidney disease.  BP, Blood pressure; VAII, Visceral adiposity and inflammation index; SD, Standard deviation. | | | | | | | | | | |

| Table S12.  Association between VAII and stroke risk by blood pressure status after excluding stroke events within the first two years of follow-up | | | | | | | | | | |
| --- | --- | --- | --- | --- | --- | --- | --- | --- | --- | --- |
| Categories | Characteristic | Event, (n%) | Model 1 | |  | Model 2 | |  | Model 3 | |
|  |  |  | HR (95% CI) | P value |  | HR (95% CI) | P value |  | HR (95% CI) | P value |
| Total | VAII  (per 1 SD) | 792 (9.2) | 1.054  (1.021, 1.089) | 0.001 |  | 1.054  (1.02, 1.089) | 0.002 |  | 1.051  (1.011, 1.091) | 0.011 |
|  | VAII |  |  |  |  |  |  |  |  |  |
|  | Q1 | 125 (5.8) | 1(Ref) |  |  | 1(Ref) |  |  | 1(Ref) |  |
|  | Q2 | 178 (8.3) | 1.475  (1.173, 1.854) | <0.001 |  | 1.477  (1.174, 1.858) | <0.001 |  | 1.409  (1.115, 1.78) | 0.004 |
|  | Q3 | 220 (10.2) | 1.849  (1.484, 2.303) | <0.001 |  | 1.846  (1.479, 2.304) | <0.001 |  | 1.737  (1.385, 2.177) | <0.001 |
|  | Q4 | 269 (12.5) | 2.35  (1.9, 2.905) | <0.001 |  | 2.383  (1.921, 2.956) | <0.001 |  | 2.108  (1.688, 2.632) | <0.001 |
|  | P for trend |  | 1.312  (1.231, 1.398) | <0.001 |  | 1.318  (1.235, 1.406) | <0.001 |  | 1.268  (1.185, 1.356) | <0.001 |
| Normal BP | VAII  (per 1 SD) | 105 (4.8) | 1.054  (0.953, 1.166) | 0.306 |  | 1.065  (0.96, 1.181) | 0.232 |  | 1.146  (0.969, 1.354) | 0.111 |
|  | VAII |  |  |  |  |  |  |  |  |  |
|  | Q1 | 20 (3.7) | 1(Ref) |  |  | 1(Ref) |  |  | 1(Ref) |  |
|  | Q2 | 25 (4.6) | 1.28  (0.711, 2.305) | 0.41 |  | 1.282  (0.711, 2.313) | 0.409 |  | 1.316  (0.726, 2.386) | 0.365 |
|  | Q3 | 31 (5.7) | 1.597  (0.91, 2.802) | 0.103 |  | 1.631  (0.926, 2.874) | 0.09 |  | 1.558  (0.881, 2.756) | 0.128 |
|  | Q4 | 29 (5.3) | 1.559  (0.882, 2.757) | 0.126 |  | 1.577  (0.887, 2.803) | 0.121 |  | 1.54  (0.856, 2.772) | 0.15 |
|  | P for trend |  | 1.161  (0.977, 1.38) | 0.09 |  | 1.167  (0.98, 1.389) | 0.083 |  | 1.151  (0.963, 1.375) | 0.122 |
| Elevated BP | VAII  (per 1 SD) | 221 (7.2) | 1.027  (0.948, 1.113) | 0.513 |  | 1.021  (0.941, 1.108) | 0.621 |  | 1.03  (0.952, 1.114) | 0.46 |
|  | VAII |  |  |  |  |  |  |  |  |  |
|  | Q1 | 42 (5.5) | 1(Ref) |  |  | 1(Ref) |  |  | 1(Ref) |  |
|  | Q2 | 46 (6) | 1.132  (0.745, 1.72) | 0.561 |  | 1.112  (0.73, 1.692) | 0.621 |  | 1.125  (0.73, 1.733) | 0.593 |
|  | Q3 | 57 (7.5) | 1.4  (0.94, 2.085) | 0.098 |  | 1.464  (0.978, 2.191) | 0.064 |  | 1.436  (0.949, 2.173) | 0.087 |
|  | Q4 | 76 (9.9) | 1.948  (1.336, 2.84) | <0.001 |  | 2.025 (1.378, 2.975) | <0.001 |  | 1.977 (1.331, 2.937) | <0.001 |
|  | P for trend |  | 1.258  (1.115, 1.419) | <0.001 |  | 1.281  (1.133, 1.449) | <0.001 |  | 1.268  (1.118, 1.438) | <0.001 |
| Hypertension | VAII  (per 1 SD) | 466 (13.8) | 1.073  (1.007, 1.143) | 0.029 |  | 1.069 (1.005, 1.137) | 0.034 |  | 1.05 (0.98, 1.124) | 0.164 |
|  | VAII |  |  |  |  |  |  |  |  |  |
|  | Q1 | 78 (9.2) | 1(Ref) |  |  | 1(Ref) |  |  | 1(Ref) |  |
|  | Q2 | 114 (13.5) | 1.494  (1.12, 1.993) | 0.006 |  | 1.477  (1.105, 1.974) | 0.008 |  | 1.372  (1.019, 1.846) | 0.037 |
|  | Q3 | 141 (16.7) | 1.835  (1.391, 2.419) | <0.001 |  | 1.849  (1.397, 2.446) | <0.001 |  | 1.745  (1.311, 2.323) | <0.001 |
|  | Q4 | 133 (15.7) | 1.815  (1.373, 2.401) | <0.001 |  | 1.854  (1.395, 2.464) | <0.001 |  | 1.691  (1.261, 2.267) | <0.001 |
|  | P for trend |  | 1.203  (1.108, 1.307) | <0.001 |  | 1.214  (1.116, 1.321) | <0.001 |  | 1.185  (1.085, 1.295) | <0.001 |
| Model 1:unadjusted for any covariates Model 2: adjusted for gender, age, residence, marital status, education level, smoking status, and drinking status Model 3: adjusted for gender, age, residence, marital status, education level, smoking, drinking, sleep time, DM, cancer, pulmonary disease, heart disease, dyslipidemia, liver disease, kidney disease.  BP, Blood pressure; VAII, Visceral adiposity and inflammation index; SD, Standard deviation. | | | | | | | | | | |

| Table S13.  Association of VAII with fall injury across blood pressure status after full-cohort multiple imputation | | | | | | | | | | |
| --- | --- | --- | --- | --- | --- | --- | --- | --- | --- | --- |
| Categories | Characteristic | Event, (n%) | Model 1 | |  | Model 2 | |  | Model 3 | |
|  |  |  | HR (95% CI) | P value |  | HR (95% CI) | P value |  | HR (95% CI) | P value |
| Total | VAII  (per 1 SD) | 2429 (15.7) | 1.029  (0.998, 1.06) | 0.064 |  | 1.021  (0.988, 1.055) | 0.208 |  | 1.006  (0.967, 1.048) | 0.758 |
|  | VAII |  |  |  |  |  |  |  |  |  |
|  | Q1 | 642 (16.6) | 1(Ref) |  |  | 1(Ref) |  |  | 1(Ref) |  |
|  | Q2 | 613 (15.8) | 1.003  (0.898, 1.12) | 0.964 |  | 0.991  (0.887, 1.108) | 0.88 |  | 0.984  (0.881, 1.1) | 0.783 |
|  | Q3 | 564 (14.6) | 0.95  (0.848, 1.063) | 0.37 |  | 0.927  (0.827, 1.039) | 0.192 |  | 0.89  (0.794, 0.998) | 0.047 |
|  | Q4 | 610 (15.7) | 1.092  (0.977, 1.22) | 0.12 |  | 1.053  (0.941, 1.178) | 0.37 |  | 0.959  (0.854, 1.076) | 0.474 |
|  | P for trend |  | 1.021 (0.985, 1.058) | 0.256 |  | 1.009  (0.973, 1.046) | 0.645 |  | 0.977  (0.942, 1.014) | 0.224 |
| Normal BP | VAII  (per 1 SD | 771 (14.8) | 1.036  (0.982, 1.094) | 0.197 |  | 1.037  (0.979, 1.099) | 0.213 |  | 1.031  (0.968, 1.099) | 0.342 |
|  | VAII |  |  |  |  |  |  |  |  |  |
|  | Q1 | 194 (14.9) | 1(Ref) |  |  | 1(Ref) |  |  | 1(Ref) |  |
|  | Q2 | 196 (15.1) | 1.05  (0.861, 1.281) | 0.627 |  | 1.041  (0.853, 1.27) | 0.694 |  | 1.017  (0.832, 1.242) | 0.873 |
|  | Q3 | 192 (14.8) | 1.098  (0.899, 1.34) | 0.36 |  | 1.09  (0.892, 1.332) | 0.401 |  | 1.068  (0.873, 1.307) | 0.521 |
|  | Q4 | 189 (14.5) | 1.157  (0.947, 1.415) | 0.154 |  | 1.13  (0.923, 1.383) | 0.24 |  | 1.075  (0.874, 1.321) | 0.495 |
|  | P for trend |  | 1.049  (0.985, 1.118) | 0.136 |  | 1.042  (0.977, 1.111) | 0.207 |  | 1.027  (0.962, 1.096) | 0.423 |
| Elevated BP | VAII  (per 1 SD) | 539 (14.8) | 0.95  (0.822, 1.099) | 0.49 |  | 0.935  (0.803, 1.088) | 0.383 |  | 0.948  (0.811, 1.109) | 0.504 |
|  | VAII |  |  |  |  |  |  |  |  |  |
|  | Q1 | 151 (16.6) | 1(Ref) |  |  | 1(Ref) |  |  | 1(Ref) |  |
|  | Q2 | 132 (14.5) | 0.987  (0.781, 1.246) | 0.91 |  | 0.938  (0.741, 1.186) | 0.592 |  | 0.938  (0.741, 1.187) | 0.592 |
|  | Q3 | 149 (16.4) | 1.075  (0.857, 1.348) | 0.531 |  | 1.05  (0.835, 1.32) | 0.678 |  | 1.013  (0.806, 1.274) | 0.913 |
|  | Q4 | 107 (11.8) | 0.781  (0.61, 1) | 0.05 |  | 0.737  (0.573, 0.948) | 0.018 |  | 0.723  (0.56, 0.932) | 0.012 |
|  | P for trend |  | 0.944  (0.876, 1.017) | 0.131 |  | 0.93  (0.861, 1.004) | 0.062 |  | 0.921  (0.853, 0.995) | 0.038 |
| Hypertension | VAII  (per 1 SD | 1119 (16.8) | 1.052  (0.996, 1.11) | 0.068 |  | 1.043  (0.985, 1.105) | 0.152 |  | 1.01  (0.949, 1.075) | 0.757 |
|  | VAII |  |  |  |  |  |  |  |  |  |
|  | Q1 | 306 (18.4) | 1(Ref) |  |  | 1(Ref) |  |  | 1(Ref) |  |
|  | Q2 | 254 (15.2) | 0.839  (0.71, 0.99) | 0.038 |  | 0.841  (0.711, 0.994) | 0.042 |  | 0.837  (0.708, 0.989) | 0.037 |
|  | Q3 | 271 (16.3) | 0.903  (0.766, 1.063) | 0.219 |  | 0.888  (0.753, 1.048) | 0.16 |  | 0.869  (0.735, 1.027) | 0.1 |
|  | Q4 | 288 (17.3) | 1.06  (0.902, 1.245) | 0.48 |  | 1.041  (0.884, 1.227) | 0.627 |  | 0.958  (0.809, 1.133) | 0.613 |
|  | P for trend |  | 1.023  (0.97, 1.079) | 0.398 |  | 1.016  (0.963, 1.072) | 0.561 |  | 0.989  (0.936, 1.045) | 0.693 |
| Model 1:unadjusted for any covariates Model 2: adjusted for gender, age, residence, marital status, education level, smoking status, and drinking status Model 3: adjusted for gender, age, residence, marital status, education level, smoking, drinking, sleep time, DM, cancer, pulmonary disease, heart disease, dyslipidemia, liver disease, kidney disease.  BP, Blood pressure; VAII, Visceral adiposity and inflammation index. | | | | | | | | | | |


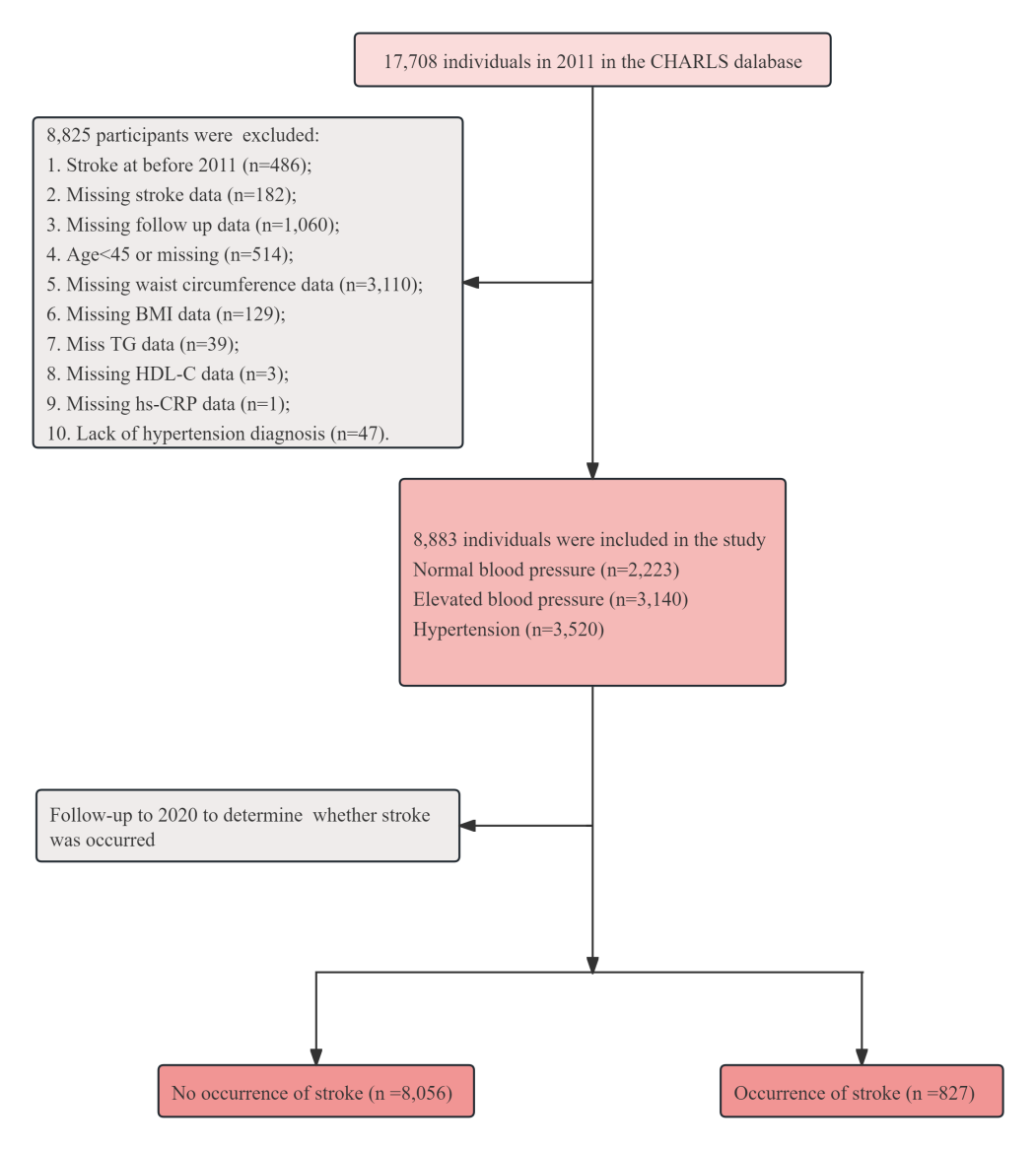


Fig. S1 Flow chart of the study population.


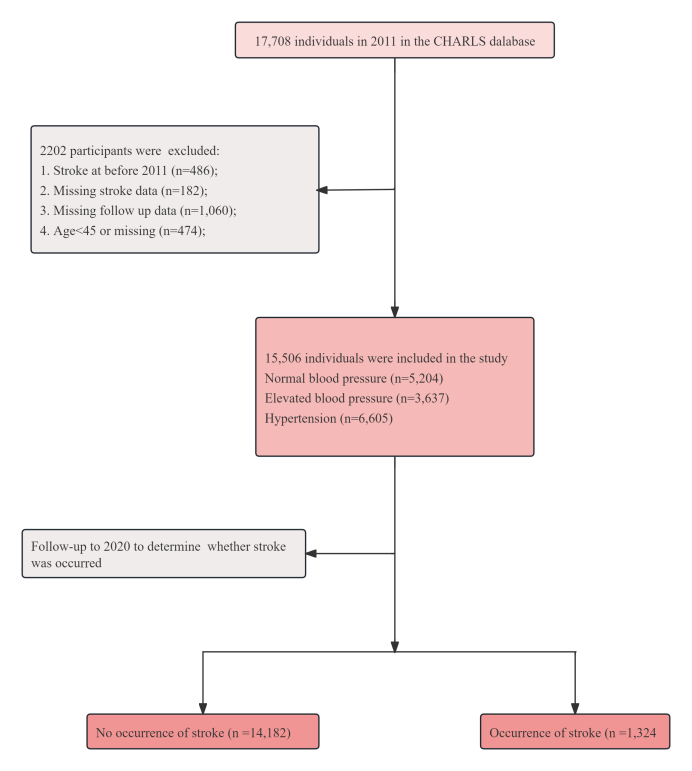


Fig. S2 Flowchart of the study population following full multiple imputation with 20 datasets in the original cohort.


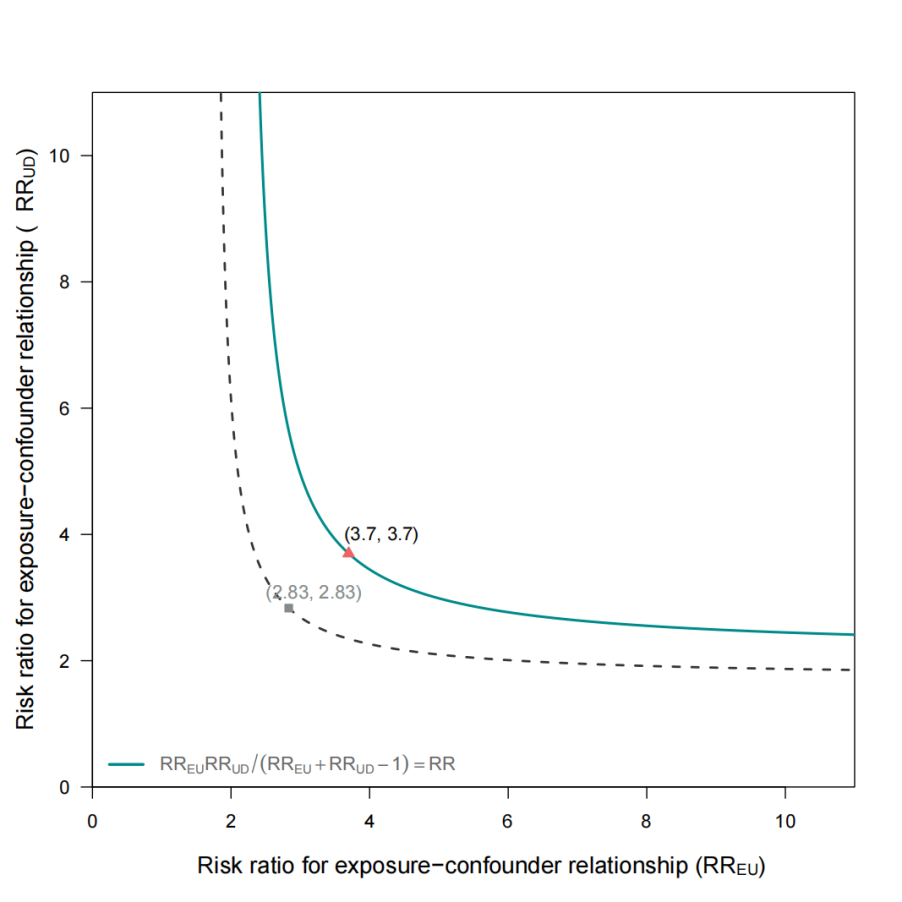


Fig. S3 E-value plot evaluating the risk ratios of VAII in relation to stroke risk. Adjusted for sex, age, residence, marital status, education level, smoking, drinking, sleep time, DM, cancer, pulmonary disease, heart disease, dyslipidemia, liver disease, kidney disease.
